# Cytoplasmic Aggregates of Splicing Factor Proline‐Glutamine Rich Disrupt Nucleocytoplasmic Transport and Induce Persistent Stress Granules

**DOI:** 10.1111/jcmm.70261

**Published:** 2024-12-05

**Authors:** Zicong Huang, Hanbin Zhang, Chuyu Huang, Runduan Yi, Xiaoyuan Zhang, Ke Ma, Wei Huang, Qingqing Wu, Yuge Zhuang, Jinsheng Liu, Wenyuan Liu, Yunhui Guo, Xiangjin Kang, Xiao Hu, Xiaochun Bai, Zhenguo Chen

**Affiliations:** ^1^ Department of Cell Biology, School of Basic Medical Sciences Southern Medical University Guangzhou Guangdong China; ^2^ Department of Obstetrics and Gynecology, Key Laboratory for Major Obstetric Diseases of Guangdong Province, Key Laboratory of Reproduction and Genetics of Guangdong Higher Education Institutes The Third Affiliated Hospital of Guangzhou Medical University Guangzhou Guangdong China; ^3^ Department of Obstetrics and Gynecology, Center for Reproductive Medicine Nanfang Hospital, Southern Medical University Guangzhou Guangdong China; ^4^ Department of key Laboratory of Oral Medicine, Guangzhou Institute of Oral Disease Stomatological Hospital of Guangzhou Medical University Guangzhou Guangdong China; ^5^ Department of Plastic and Burn Surgery Guangzhou Red Cross Hospital (Guangzhou Red Cross Hospital of Jinan University) Guangzhou Guangdong China; ^6^ Department of Anesthesiology Guangdong Provincial People's Hospital (Guangdong Academy of Medical Sciences), southern Medical University Guangzhou Guangdong China

**Keywords:** cytoplasmic aggregate, nucleocytoplasmic transport, RNA metabolism, SFPQ, stress granule

## Abstract

Splicing factor proline‐glutamine rich (SFPQ), a multifunctional RNA‐binding protein (RBP), shows cytoplasmic colocalisation with stress granule (SG) markers; however, the causative relationship and mechanism underlying this coalescence of SFPQ aggregates and SGs remain unclear. In this study, we demonstrate that SFPQ lacking its nuclear localisation sequence spontaneously forms cytoplasmic aggregates that abnormally incorporate immature RNA and induce persistent SGs. mRNA profiling showed that SFPQ mislocalisation induced extensive changes in RNA processing, with a subset of alternatively spliced transcripts associated with nucleocytoplasmic transport. Notably, these altered transporters were sequestered into SFPQ aggregates, constituting aberrant protein‐RNA complexes. Importantly, suppression of SG nucleation could not block cytoplasmic SFPQ aggregation with immature RNA and nucleocytoplasmic transporters, both of which, however, were moderately ameliorated by the inhibition of alternative splicing or nuclear export. Our results unveil the physiopathological role and mechanism for mislocalised SFPQ in the RNA metabolism, nucleocytoplasmic transport and pathological SGs.

## 1 Introduction

Stress granules (SGs) are a type of discrete cytoplasmic foci composed of mRNAs, RNA‐binding proteins (RBPs), small ribosomal subunits and eukaryotic initiation factors (eIFs) [1, 2], and the formation of SGs is an important stress response mechanism in response to environmental insults [3]. When SGs assemble, multiple RNA–RNA, RNA–protein and protein–protein interactions coalesce under proteotoxic stress conditions after the global stalling of protein translation induced by liquid–liquid phase separation into small granules [4, 5]. Once the instigating stress resolves, SGs dissolve and mRNA translation resumes. To date, the molecular network of SGs has been further examined, thanks to the application of proximity labeling [3, 6, 7], but further research is still required to fully understand the molecular pathways underlying the dynamics of SGs assembly and disassembly.

Although SGs were initially considered to promote cell survival during adverse conditions, defects in SG formation or clearance have been found to be highly implicated in a variety of diseases, including neurodegeneration, cancer, diabetes and autoimmune disease [8]. Previous research has indicated that persistent stress, which is linked to neurodegenerative diseases, causes RBPs in the cytoplasm to form pathological SGs, which eventually develop into amyloids, abnormal clumps of nonfunctional proteins [9, 10, 11]. For example, TAR DNA binding protein 43 (TDP‐43) has been found to be depleted from the nucleus and to accumulate as large cytoplasmic aggregates, and subsets of these inclusions colocalise with SG markers in the degenerating neurons and glia of these patients [12, 13, 14]. Like TDP‐43, fused in sarcoma (FUS) protein is normally a nuclear protein, and ALS‐causing mutations result in its cytoplasmic inclusion, with such inclusions testing positive for SG markers [13, 15, 16]. In addition, the connections between SG assembly and nucleocytoplasmic transport remain unclear. Importantly, nucleocytoplasmic transport defects have also been identified as a critical event in the pathogenesis of neurodegenerative diseases, such as ALS/FTLD [17, 18, 19], Huntington's disease [20] and Alzheimer's disease [21]. These reports imply that disruption of nucleocytoplasmic transport contributes to cytoplasmic RBP aggregates and SGs becoming more closely connected, structurally and/or functionally. What has remained elusive, however, is the causative relationship among RBP dysfunction, nucleocytoplasmic transport disorders, pathological SGs and neurodegeneration.

Polypyrimidine tract‐binding protein‐associated splicing factor/splicing factor proline‐glutamine rich (PSF/SFPQ) is a multifunctional RBP, with diverse roles in alternative splicing, transcriptional regulation, RNA processing and paraspeckle formation [22, 23]. SFPQ has recently been found to be associated with cytoplasmic RNA granules and is required for axonal trafficking of mRNAs [24, 25, 26]. Importantly, several lines of evidence have demonstrated that SFPQ is implicated in neurodegenerative diseases, and it has been found to aberrantly translocate to the cytoplasm and form inclusions with SG proteins [10, 27, 28, 29]. However, there are several important questions that remain unanswered: (1) what is the causative relationship between cytoplasmic SFPQ aggregates and persistent SGs; (2) how do cytoplasmic SFPQ aggregates associate with SGs and affect SG dynamics; (3) what are the contributions of RNA processing and nucleoplasmic transport to the SFPQ‐associated persistent SGs; and (4) can inhibition of SG assembly or nucleoplasmic transport rescue SFPQ cytoplasmic mislocalisation and its associated impacts; or attenuating SFPQ mislocalisation prevent pathological SGs?

To address these issues, we generated a truncated mutant of SFPQ by deleting its C‐terminal nuclear localisation sequence (ΔNLS, Δ amino acid [aa] 701–707) [23, 30, 31], which replicates the cytoplasmic mislocalisation of SFPQ in cells. We show that cytoplasmic mislocalisation of SFPQ formed cytoplasmic aggregates under physiological conditions, which tightly associated with SG components and greatly disturbed SG dynamics. Importantly, cytoplasmic mislocalisation of SFPQ induced severe abnormalities in RNA metabolism, characterised by aberrant splicing of nucleocytoplasmic transporters and mislocalisation of these transporters and RNA into cytoplasmic SFPQ aggregates concurrent with persistent SGs, leading to detrimental RNA–protein complexes. Altogether, our findings link the cytoplasmic accumulation of SFPQ, nucleocytoplasmic transport deficiency and pathogenic SGs in a unified pathway.

## 2 Materials and Methods

### 2.1 Constructs

All plasmids were constructed by Genechem Co. Ltd. (Shanghai, China). Flag‐UBAP2L plasmid was generated in our previous work [32]. The cDNA for human SFPQ (NM_001536) was synthesised and cloned into the pcDNA3.1 vector with a Myc tag. This plasmid (full length, Myc‐SFPQ‐WT) was the seed for further generation of Myc‐tagged SFPQ truncations: △701‐707, deleting aa 701‐707 containing the C‐terminal NLS domain, △701‐707 plus △1‐27 (lacking both the C‐terminal NLS and the RGG motif), △701‐707 plus △299‐449 (lacking the C‐terminal NLS and the two RNA‐recognition motif (RRM)) and △498‐707 (lacking both the two NLSs and the coiled‐coil region rich in Gly and Pro). Adenovirus harbouring Flag tag, Flag‐SFPQ‐WT or Flag‐SFPQ△701‐707 were generated by the OBiO Technology Co. Ltd. (Shanghai, China).

### 2.2 Animals

The 5 × FAD (five‐fold Familial Alzheimer's Disease) mice, an Alzheimer's disease mouse model, on a congenic C57BL/6J background were kindly provided by Prof. Xin‐Hong Zhu (Southern Medical University). The 5 × FAD mice carry three APP mutations (Swedish [K670N/M671L], Florida [I716V] and London [V717I]) and two PSEN1 mutations (M146L and L286V), all of which are known to be related to familial Alzheimer's disease [33, 34, 35]. All animal experiments were approved by the Ethics Board of Southern Medical University. Mice were housed in a temperature‐ and humidity‐controlled room with a 12‐h light/12‐h dark cycle with ad libitum access to water and a standard laboratory diet. Hemizygous 6‐month‐old male and female transgenic mice and their non‐transgenic littermates were used.

### 2.3 Cell Culture and Transfections

HEK293T and SH‐SY5Y cells were maintained in a 5% CO_2_, 37°C‐humidified incubator, and cultured in Dulbecco's Modified Eagle Medium (DMEM) (Corning, NY, USA) supplemented with 10% fetal bovine serum (FBS) (AusGeneX, Brisbane, Australia). siRNA or plasmids were transfected into HEK293T cells using Lipofectamine 3000 (Invitrogen, Carlsbad, CA, USA). After 48 h, the cells were treated with SG‐inducing chemicals, followed by chemical removal, and subjected to further analyses. siRNA sequences targeting *SFPQ* were as follows: sense 5′‐CCAGAAGAAUCCAAUGUAUTT‐3′, antisense 5′‐AUACAUUGGAUUCUUCUGGGC. siRNA sequences targeting *G3BP1* were as follows: sense 5′‐CAUUAACAGUGGUGGGAAATT‐3′, antisense 5′‐UUUCCCACCACUGUUAAUGTT. siRNA sequences targeting *G3BP2* were as follows: sense 5′‐UGAAGGAUCUGUUCCAAAUTT‐3′, antisense 5′‐AUUUGGAACAGAUCCUUCATT. SH‐SY5Y cells were transfected with an adenoviral Flag tag, Flag‐SFPQ‐WT or Flag‐SFPQ△701‐707 and then subjected to the indicated treatments and analyses including RNA sequencing.

### 2.4 Chemical Reagents, Treatments and SG Dynamics

Chemical reagents used for SG induction included sodium arsenite (AS), H_2_O_2_, sorbitol (all from Sigma‐Aldrich, Shanghai, China) and NaCl (Guangzhou Chemical Reagent Co. Ltd., Guangzhou, China). SGs were induced by treatment with AS (500 μM for 1 h), H_2_O_2_ (1 mM for 1 h), NaCl (200 mM for 30 min) or sorbitol (400 mM for 30 min). AS‐treated cells were further recovered from stress at 1, 2, 4, 8 and 12 h to monitor SG disassembly. Cells were scored for SGs by counting the microscopically visible granules with UBAP2L, G3BP or G3BP2 as the SG marker, and the size of SGs was assessed by measuring the fluorescence intensity as described previously [32], using Image J software (v1.52a, National Institutes of Health, Bethesda, MD, USA). In some experiments, cells were treated with KPT‐330 (10 μM, Selleck Chemicals, Shanghai, China) for 24 h to suppress nucleocytoplasmic transport or with PlaB (Santa Cruz Biotechnology, Dallas, TX, USA) for 6 h to inhibit splicing. In the experiments with Myc‐ or Flag‐tagged SFPQ deletants, only Myc‐ or Flag‐positive cells were counted. At least 100 cells from at least five fields were analysed.

### 2.5 Immunofluorescence

Cells were planted on glass bottom confocal dishes, treated as indicated, fixed in 4% paraformaldehyde, and permeabilised by 0.1% Triton X‐100. Cells were blocked in 5% normal goat serum and then incubated with primary antibodies overnight at 4°C, followed by a secondary incubation with Alexa‐Fluor 488‐ or 594‐labelled secondary antibodies (Jackson Immunoresearch, West Grove, PA, USA). We used 4, 6‐diamidino‐2‐phenylindole (D9542, Sigma‐Aldrich) to visualise the nuclei.

For the staining of brain tissues, samples were first fixed with 4% formaldehyde in phosphate buffered saline (PBS). After subsequent dehydration with an ethanol gradient, tissues were embedded in paraffin and sectioned into 5 μm sections and then deparaffinised and dehydrated through a graded ethanol series. The sections were repaired in citrate buffer under high pressure for 5 min, followed by 5 min in PBS, and incubated with 5% bovine serum albumin (BSA) for 1 h. After incubation with primary antibodies and Alexa‐Fluor‐488‐or Alexa‐Fluor‐594‐labelled secondary antibodies, nuclei were stained with DAPI.

Immunofluorescent images were obtained using a FluoView FV1000 confocal microscope (Olympus, Tokyo, Japan) with a 20×, 40× or 60× objective. All images are single plane images, with each image corresponding to an optimal focal plane. The laser light sources include 405, 488 and 543 nm, which are used to excite different fluorescent labels. The same set of images were captured under the same exposure time and laser power to ensure consistency and comparability. The primary antibodies used for IF staining are listed in Table S1.

### 2.6 Western Blotting

Protein lysates were subjected to 6%–12% sodium dodecyl sulfate‐polyacrylamide gel electrophoresis (SDS‐PAGE) and electrotransferred to nitrocellulose membranes (GE Healthcare Life Sciences, Beijing, China). The membranes were then blocked in 5% nonfat dry milk and incubated with primary antibodies overnight at 4°C. After incubation with horseradish peroxidase‐conjugated secondary antibodies (Jackson Immunoresearch), protein bands were finally visualised using an enhanced chemiluminescence kit (PerkinElmer, Waltham, MA, USA). The primary antibodies used for western blot (WB) analysis are listed in Table S1.

### 2.7 Co‐Immunoprecipitation (IP)

IP assays were performed as described previously [32]. Briefly, treated cells were lysed in cold CHAPS‐containing lysis buffer (0.3% CHAPS, 40 mM HEPES [pH 7.4], 150 mM NaCl, 2 mM ethylenediamindium pyrophosphate, 10 mM sodium glycerophosphate, 50 mM NaF, plus protease inhibitors). In some experiments, 40 μg/mL RNase A with/without EDTA‐EGTA (EE; 2 mM EDTA, 2.5 mM EGTA) or 5 mM MgCl_2_ were added into the lysis buffer. Cells were rotated at 4°C for 20 min, centrifuged at 9700× *g* at 4°C for 10 min, and incubated with primary antibodies at 4°C for 2 h with continuous rotation. A 50% slurry of protein G Sepharose (60 μL, Cytiva, Uppsala, Sweden) was then added and incubated for an additional 1 h. Immunoprecipitated proteins were denatured by addition of 50 μL of SDS loading buffer and boiling for 5 min, resolved by SDS‐PAGE, and finally analysed by immunoblotting.

For mass spectrometry (MS) analysis, immunoprecipitated proteins were subjected to trypsin digestion, high‐performance liquid chromatography‐MS/MS, and data analysis by Fitgene Biotechnology Co. Ltd. (Guangzhou, China), as detailed in supporting methods (Appendix S6).

### 2.8 Newborn RNA Labelling by 5′‐EU Incorporation

To label newborn RNA, cells were cultured in DMEM containing 500 μM 5′‐EU for 6 h at 37°C before fixation. Incorporated EU was detected using the Cell‐Light EU Apollo 488 In Vitro Imaging Kit (C10316‐3, RiboBio, Guangzhou, China), according to the manufacturer's protocol.

### 2.9 Cytoplasmic and Nuclear Protein Extraction

Cytoplasmic and nuclear protein extraction assays were performed as described previously [32], using a commercial kit (Invent Biotechnologies, Beijing, China). Briefly, confluent HEK293T cells plated in a 6‐well plate were treated as indicated, rinsed with cold PBS and then lysed in cytoplasmic extraction buffer on ice for 5 min. Cell lysates were transferred to pre‐chilled 1.5 mL tubes, which were vortexed vigorously for 15 s. After centrifugation at 18,000 *g* and 4°C for 5 min, the supernatant was collected and stored as the cytosolic fraction. After addition of appropriate amounts of nuclear extraction buffer to the pellet, the solution was vortexed vigorously for 15 s and incubated on ice for 1 min. Vorexing for 15 s and incubation for 1 min were repeated four times. After centrifugation at 4°C and 18,000 *g* for 30 s, nuclear extracts were collected for further analysis.

### 2.10 RNA Extraction and Quantitative Real‐Time Polymerase Chain Reaction (PCR)

RNA was purified using Trizol reagent (Invitrogen) and processed to cDNA using the Hifair II 1st Strand cDNA Synthesis Kit, followed by amplification and quantification using Hieff qPCR SYBR Green Master Mix (all from YEASEN Biotech Co. Ltd., Shanghai, China) with a StepOne Plus Real‐Time PCR System (Applied Biosystems, Waltham, MA, USA). *Gapdh* was used as the endogenous control transcript. Three technical replicates were performed for each transcript. The primer sequences are shown in Table S2.

### 2.11 RNA Sequencing and Data Analysis

RNA preparation, transcriptome sequencing and data analysis were all performed by Novogene (Beijing, China). RNA sequencing data have been deposited in the NCBI database (PRJNA886326). Detailed protocols are presented in supporting methods (Appendix S6).

### 2.12 Statistical Analysis

Quantification and statistical analyses were carried out in Microsoft Excel and performed using GraphPad Prism 8 software (GraphPad Software, San Diego, CA, USA). *p* < 0.05 was considered statistically significant. Statistical tests are described in the figure legends.

## 3 Results

### 3.1 SFPQ is a Novel UBAP2L Binding Partner but Barely Associates With Normal SGs

**FIGURE 1:**
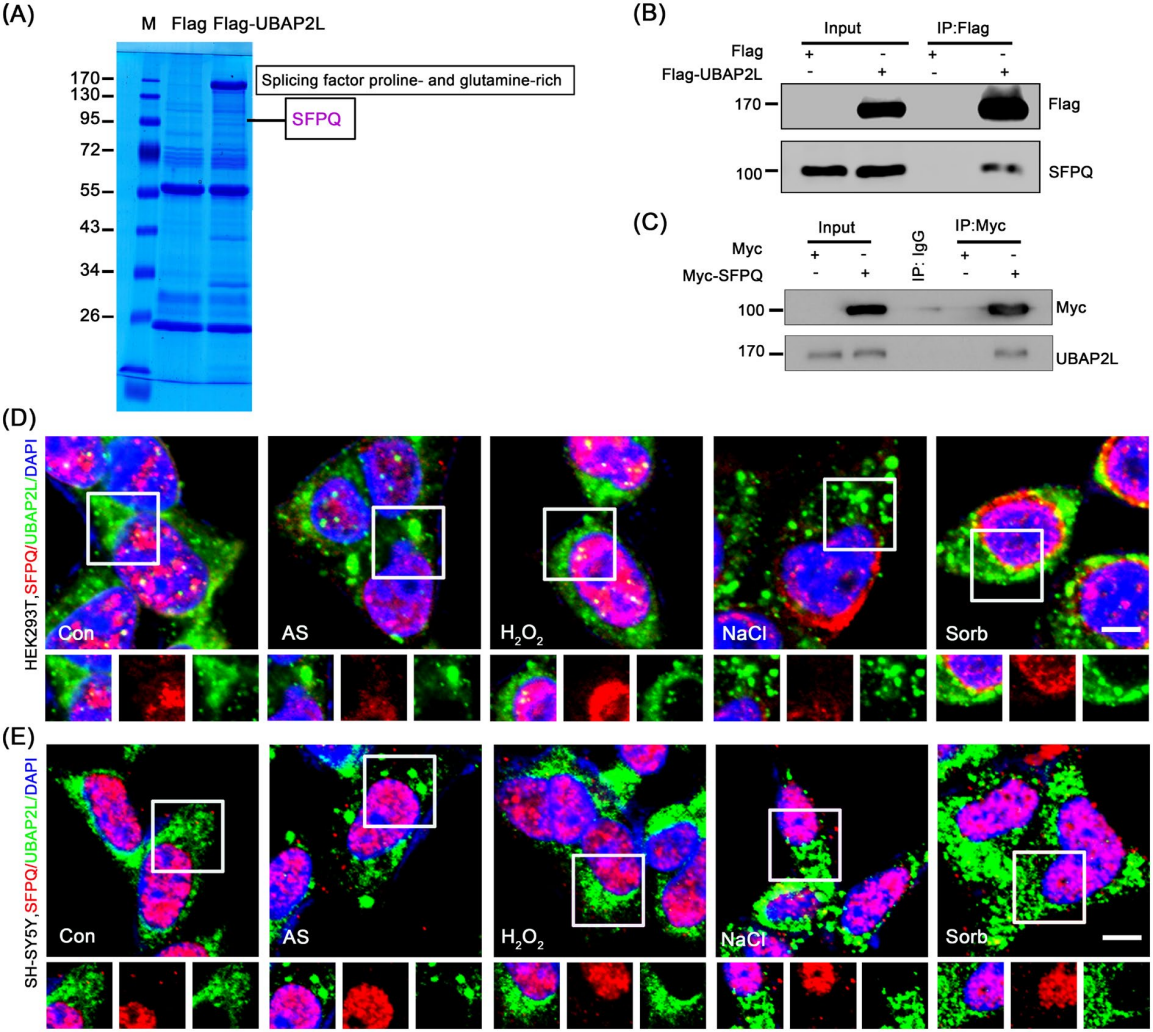
SFPQ is a novel binding partner of UBAP2L but hardly associates with SGs. (A) Coomassie blue‐stained gel showing SFPQ interacting with UBAP2, identified by an IP/MS assay. HEK293 cells were transfected with the Flag tag or Flag‐UBAP2L plasmids. After 48 h, IP was performed using a Flag primary antibody coupled with an MS assay. M, marker. (B, C) Validation of the interaction between SFPQ and UBAP2L by reciprocal IP. HEK293 cells were transfected with Flag‐UBAP2L or Myc‐SFPQ plasmids, and reciprocal IP was performed using a Flag or Myc primary antibody, respectively, followed by a WB analysis of endogenous SFPQ or UBAP2. (D) Co‐immunofluorescence of SFPQ (red)/UBAP2L (green) under physiological or stress conditions. HEK293T cells were treated with or without 500 μM sodium arsenite (AS) or 1 mM H_2_O_2_ for 1 h, or 400 mM sorbitol (sorb) or 200 mM NaCl for 30 min, and then stained for SFPQ (red)/UBAP2L (green). Insets are shown with separated colours. Blue indicates the nuclei counterstained by DAPI. (E) Co‐immunofluorescence of SFPQ (red)/UBAP2L (green) in SH‐SY5Y cells. The treatments were the same as in (D). All scale bars: 5 μm.

Mislocalisation of SFPQ from the nucleus to the cytoplasm results in inclusions with SG proteins; however, the role of SFPQ in governing SG assembly has not been fully elucidated. When we documented the role and mechanism of ubiquitin‐associated protein 2‐like (UBAP2L) in SG dynamics [32], IP/MS analysis revealed that SFPQ was present in precipitates from the Flag‐labelled UBAP2L group but not in immune‐precipitates labelled with the Flag tag only (Figure 1A). Mascot search results showed that the peptides correlated with the protein sequences of SFPQ ( Supplementary Text 1 in Appendix S6). Further IP/WB analysis confirmed that ectopic UBAP2L precipitated endogenous SFPQ (Figure 1B), and ectopic SFPQ also associated with endogenous UBAP2L (Figure 1C). These results demonstrated that SFPQ binds to UBAP2L. To further verify this molecular interaction and examine the possible association of SFPQ with SGs, HEK293T and SH‐SY5Y cells were exposed to various chemicals to induce SGs, followed by co‐immunofluorescence (IF) staining of SFPQ and UBAP2L. Under physiological conditions, SFPQ was predominantly localised to the nucleus of HEK293T cells, with weak signals distributed in the cytoplasm (Figure 1D). HEK293T cells formed larger clear UBAP2L‐positive granules after exposure to sodium arsenite (AS) than under H_2_O_2_, NaCl or sorbitol conditions. However, SFPQ did not evidently colocalise with UBAP2L‐positive granules, and no stress treatment induced significant nucleocytoplasmic translocation of SFPQ (Figure 1D). Similar results were observed in SH‐SY5Y cells (Figure 1E). IF staining of SFPQ and G3BP2, another SG marker, also showed similar results to those of UBAP2L in both cells (Figure S1A,B). In line with the IF data, WB analyses showed that stress treatment or recovery did not alter the protein level of SFPQ (Figure S1C), and SFPQ knockdown also did not affect the expression of other SG proteins (Figure S1D). These data together suggested that SFPQ is predominantly distributed in the nucleus, but barely translocates into the cytoplasm and does not associate with normal UBAP2L‐ or G3BP2‐positive SGs upon stress.

### 3.2 Ectopic Cytoplasmic SFPQ Associates With SGs and Alters SG Disassembly Dynamics

**FIGURE 2:**
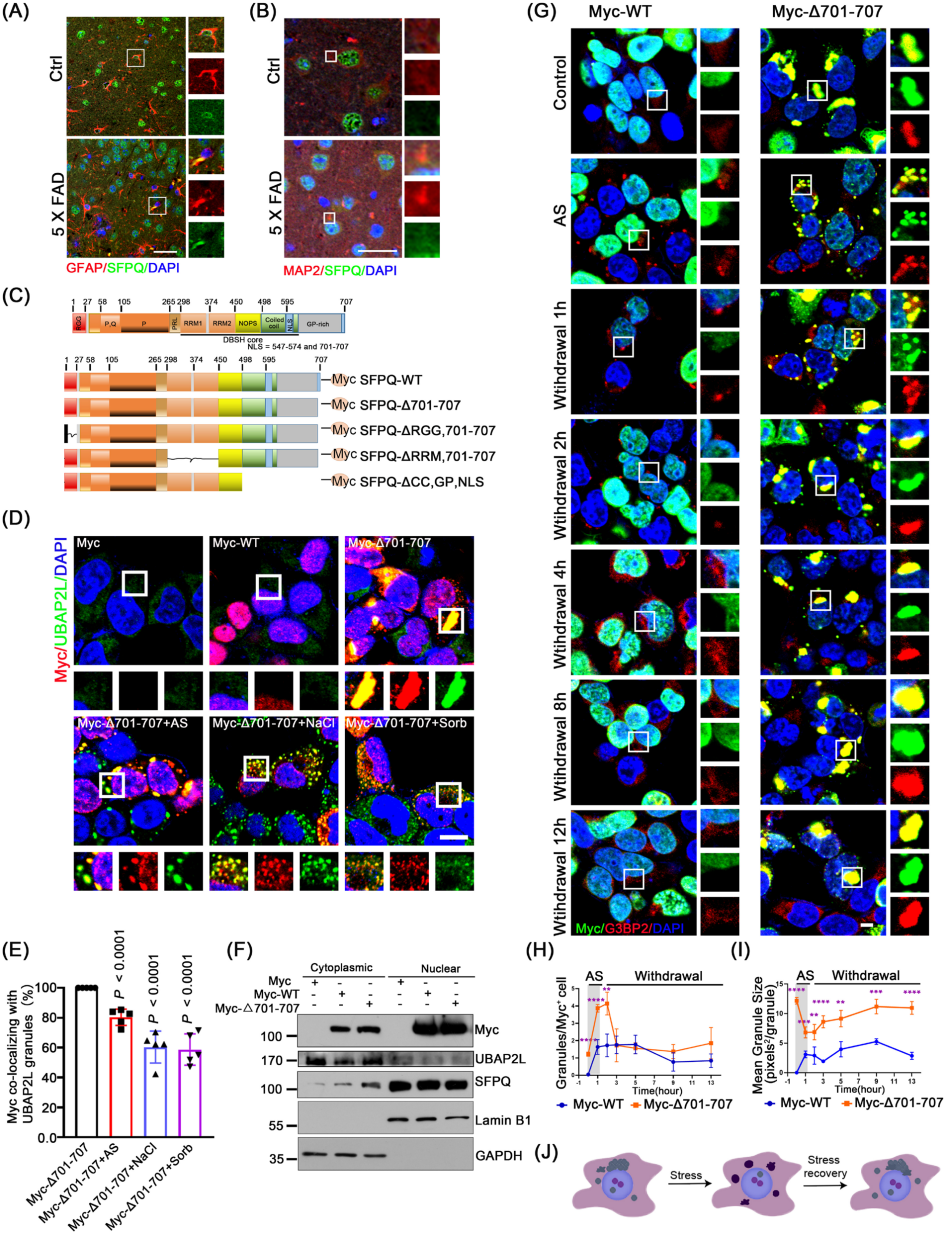
Ectopic cytoplasmic SFPQ forms aggregates and disturbs SG disassembly dynamics. (A) Colocalisation of GFAP (red, marker of astrocyte) and SFPQ (green) in the cortex sections of 6‐month‐old 5 × FAD mice. Scale bars: 20 μm. (B) Colocalisation of MAP2 (red, marker of neuron) and SFPQ (green) in the cortex sections of 6‐month‐old 5 × FAD mice. Scale bars: 20 μm. (C) Schematics of SFPQ wild‐type (WT) and its deletants. RGG, Arg‐Gly‐Gly; RRM, RNA‐recognition motifs; CC, coiled‐coil; GP, Gly and Pro; NLS, nuclear location sequence. ΔRGG,701–707, deleting aa 1–27 and aa 701–707; ΔRRM, deleting aa 297–452 and aa 701–707; ΔCC, GP, NLS, deleting aa 498–707. (D) Colocalisation of SFPQ deletants with UBAP2L in HEK293T cells expressing Myc empty, Myc‐WT or Myc‐Δ701‐707 with or without 500 μM AS for 1 h, or 400 mM sorbitol or 200 mM NaCl for 30 min, followed by co‐fluorescence of Myc (red)/UBAP2L (green). Nuclei were counterstained with DAPI (blue). (E) Percentages of cytoplasmic Myc foci colocalised with UBAP2L granules in Myc^+^ cells in (D). Images are representative of 5 independent frames for each condition, and 20–35 granules were analysed per condition. Data are the mean ± standard deviation (SD) and represent three biologically independent experiments. *P* values were determined using one‐way ANOVA with Tukey's multiple comparisons test, comparing Myc‐△701‐707 to others treatments. (F) Cytoplasmic and nuclear protein fractionation assay for the expression of Myc and SFPQ in HEK293T cells expressing Myc, Myc‐WT or Myc‐Δ701‐707. GAPDH and Lamin B1 were used as cytoplasmic and nuclear markers, respectively. (G) G3BP2 dynamics by IF. HEK293T cells expressing Myc‐WT or Myc‐Δ701‐707 were treated with AS (500 μM, 1 h) or relieved from AS for 1 h to 12 h, followed by IF of Myc (green)/G3BP2 (red). (H) Line chart showing changes in the number of G3BP2 granules visualised in (G), Only Myc‐positive cells were analysed. One‐way analysis of variance (ANOVA), comparing Myc‐WT to Myc‐Δ701‐707, with Dunnett correction for multiple testing: ****p < 0.0001, **p = 0.0032, NS, not significant (*p* > 0.05), *n* = 4. (I) Line chart showing changes in the size of G3BP2 granules visualised in (G), *n* = 4, one‐way analysis of variance (ANOVA), comparing Myc‐WT to Myc‐Δ701‐707, with Dunnett correction for multiple testing: ****p < 0.0001, ***p = 0.0005, **p = 0.0068, ****p < 0.0001, **p = 0.0016, ***p = 0.0001, ****p < 0.0001. Granular size is expressed as pixels^2^ per granule. Data are the mean ± standard deviation (SD). (J) Schematic depicts that mislocalised SFPQ disrupts SG disassembly dynamics. All scale bars: 5 μm.

Previous studies have identified cytoplasmic localisation of SFPQ in Alzheimer's disease patients, along with its colocalisation with p‐Tau. This suggests a potential involvement of ectopic cytoplasmic SFPQ in SG formation. To investigate whether SFPQ is aberrantly transported into the cytoplasm of brain cells under pathological conditions, we examined the colocalisation of SFPQ with glial fibrillary acidic protein (GFAP, an astrocyte‐specific marker) and microtubule‐associated protein 2 (MAP2, a neuron‐specific marker) in brain tissue slices from 6‐month‐old 5 × FAD mice, an Alzheimer's disease mouse model. Compared with WT mouse brains, SFPQ was translocated from the nucleus to the cytoplasm in both astrocytes (Figure 2A) and neurons (Figure 2B) in the 5 × FAD mice. Importantly, it was previously indicated that SFPQ contains two NLSs that are both required for complete transport into the nucleus (Figure 2C), and neither of these NLSs can independently bestow complete nuclear localisation [31]. To investigate the association between the cytoplasmic dislocation of SFPQ and SGs, we next generated an artificially engineered SFPQ by deleting the C‐terminal NLS (aa 701–707), with all other functional domains being preserved (SFPQΔ701‐707; Figure 2C). When transfected into HEK293T cells, ectopic wild‐type SFPQ (SFPQ‐WT) was prevailingly restricted within the nucleus, while SFPQΔ701‐707 was partially excluded from the nucleus and distributed in the cytoplasm; surprisingly, SFPQ automatically formed notable and large cytoplasmic aggregates, where it colocalised with UBAP2L (Figure 2D,E) and G3BP2 even without stress (Figure S2B). Upon stress, more UBAP2L‐ or G3BP2‐positive granules were formed, and notably, all of these granules were enclosed in SFPQ aggregates in SFPQΔ701‐707 cells (Figure 2D,E, Figure S2B), while this phenomenon was not observed in the SFPQ‐WT group (Figure S2A). In SH‐SY5Y cells, SFPQΔ701‐707 showed weak and dispersed distribution in the cytoplasm and did not form clear aggregates under physiological conditions (Figure S2C,D). Accordingly, SFPQΔ701‐707 showed weak colocalisation with G3BP (Figure S2C) or UBAP2L (Figure S2D) under physiological conditions and associated with UBAP2L or G3BP2 granules notably. This may indicate the differential requirement of the aa 701–707 NLS between these two cells. We suggest that this automatic aggregation of cytoplasmic SFPQ is a general mechanism, as HeLa cells with SFPQΔ701‐707 showed similar performance (Figure S2E). Altogether, these data suggested that loss of the aa 701–707 NLS could trigger the cytoplasmic mislocation of SFPQ under stress conditions, where it spontaneously forms clear cytoplasmic aggregates and tightly associates with SG proteins.

To examine the impact of ectopic SFPQΔ701‐707 on endogenous SFPQ expression, we then separated the cytoplasmic and nuclear components of HEK293T cells expressing SFPQΔ701‐707, followed by WB analyses of SFPQ (Figure 2F). The findings showed that SFPQ was mostly expressed in the nucleus, with only weakly detectable SFPQ signals seen in the cytoplasm. Ectopic SFPQ‐WT resulted in modestly elevated SFPQ expression in the cytoplasm. Ectopic SFPQ△701‐707 most efficiently increased SFPQ in both the cytoplasm and the nucleus and did not induce nuclear deletion of endogenous SFPQ. These data suggested that the observed cellular phenotypes were predominantly induced by cytoplasmic mislocalisation.

Besides the NLS, SFPQ contains several important domains, including an Arg‐Gly‐Gly (RGG) box (aa 1–27), two RNA‐recognition motifs (RRM1, aa 299–369 and RRM2, aa 370–449) and a coiled‐coil region enriched for Gly and Pro (aa 498–700; Figure 2C). These features confer SFPQΔ701‐707 with the ability to nucleate SG formation. We tested whether loss of these domains further in SFPQΔ701‐707 (Figure 2C) affected the association with SGs by IF. As shown in Figure S3A,B, additional loss of each domain made SFPQΔ701‐707 more dispersed around the cytoplasm and therefore less associated with UBAP2L foci. Particularly, deletion of the RRM most significantly decreased the number and size of UBAP2L foci. This observation indicated that the RRM plays a pivotal role in SFPQΔ701‐707 recruiting RNA or SG proteins for potential nucleation, which further supports the critical role of the RRM motifs in SG formation [9, 36, 37].

Having observed that cytoplasmic SFPQ formed aggregates and tightly associated with SGs, we next examined whether cytoplasmic SFPQ interferes with SG dynamics. HEK293T cells were transfected with Myc‐SFPQ‐WT or Myc‐SFPQΔ701‐707 plasmid and then were stressed with AS for 1 h, followed by recovery for 1 to 12 h. Only G3BP2 granules in the cells with a positive Myc signal were monitored. G3BP2 was incorporated into the SFPQΔ701‐707 aggregates and displayed a granule‐like configuration under physiological conditions (Figure 2G). When stressed with AS, both the number and size of G3BP2 granules were markedly increased in the SFPQΔ701‐707 group, compared with the SFPQ‐WT group. Upon AS removal, G3BP2 granules in the SFPQ‐WT group dissolved normally, and by 8 h, almost no visible granules were observed (Figure 2G–I). By contrast, in the SFPQΔ701‐707 group, the number of G3BP2 granules was rapidly reduced, while their size increased gradually during the first 2 h, and thereafter remained steady (Figure 2G–I). These observations suggested that in the presence of cytoplasmic SFPQΔ701‐707 aggregates, G3BP2 formed granule‐like accumulations and then converted into multiple smaller SGs upon stress. These smaller SGs were prone to coalesce into a large compact SG again after removal of the stressor and failed to dissolve (Figure 2J). These interesting observations led to the conclusion that ectopic cytoplasmic SFPQ induces the accumulation of SG proteins and disturbs SG disassembly dynamics, thus contributing to pathogenic SGs.

### 3.3 Mislocalised Cytoplasmic SFPQ Induces Cell Injury

To explore the effect of cytoplasmic ectopia of SFPQ on cellular function, and whether these changes correlated with neurodegeneration, SH‐SY5Y cells were transfected with adenovirus carrying a Flag tag only, Flag‐SFPQ‐WT or Flag‐SFPQΔ701‐707. We then performed mRNA sequencing, and the data obtained for the Flag‐SFPQ‐WT and Flag‐SFPQΔ701‐707 groups were adjusted, using the Flag tag group as a baseline control. The SFPQ‐WT group exhibited 604 differentially expressed genes (DEGs, 312 upregulated, 292 downregulated), and the SFPQ701–707 group exhibited 478 DEGs (172 upregulated, 306 downregulated, Appendix S1). Kyoto Encyclopedia of Genes and Genomes analyses of the sequencing data showed that the upregulated DEGs in SFPQ△701‐707 were mostly enriched in the pathways related to neurodegenerative diseases, including Alzheimer's, Parkinson's and Huntington's diseases (Figure S3C, Appendix S2), suggesting the implication of mislocalised cytoplasmic SFPQ in neurodegenerative diseases. Neurodegeneration is commonly caused by neuronal death. We then investigated whether cytoplasmic SFPQ mislocalisation results in cell injury. WB analyses showed a significant rise in the protein level of pro‐apoptotic cleaved‐PARP in SH‐SY5Y cells with SFPQΔ701‐707 (Figure S3D,E). IF staining of γH2AX, a marker for DNA breakage, also indicated that SH‐SY5Y cells with SFPQΔ701‐707 suffered more DNA damage (Figure S3F,G). Similar results were also observed in HEK293T cells (Figure S3H‐K). These observations together suggested that mislocalised cytoplasmic SFPQ produced pathological SGs and led to cell injury.

### 3.4 Mislocalised Cytoplasmic SFPQ Precipitates SG Components and Causes Abnormal Nucleocytoplasmic RNA Export

**FIGURE 3:**
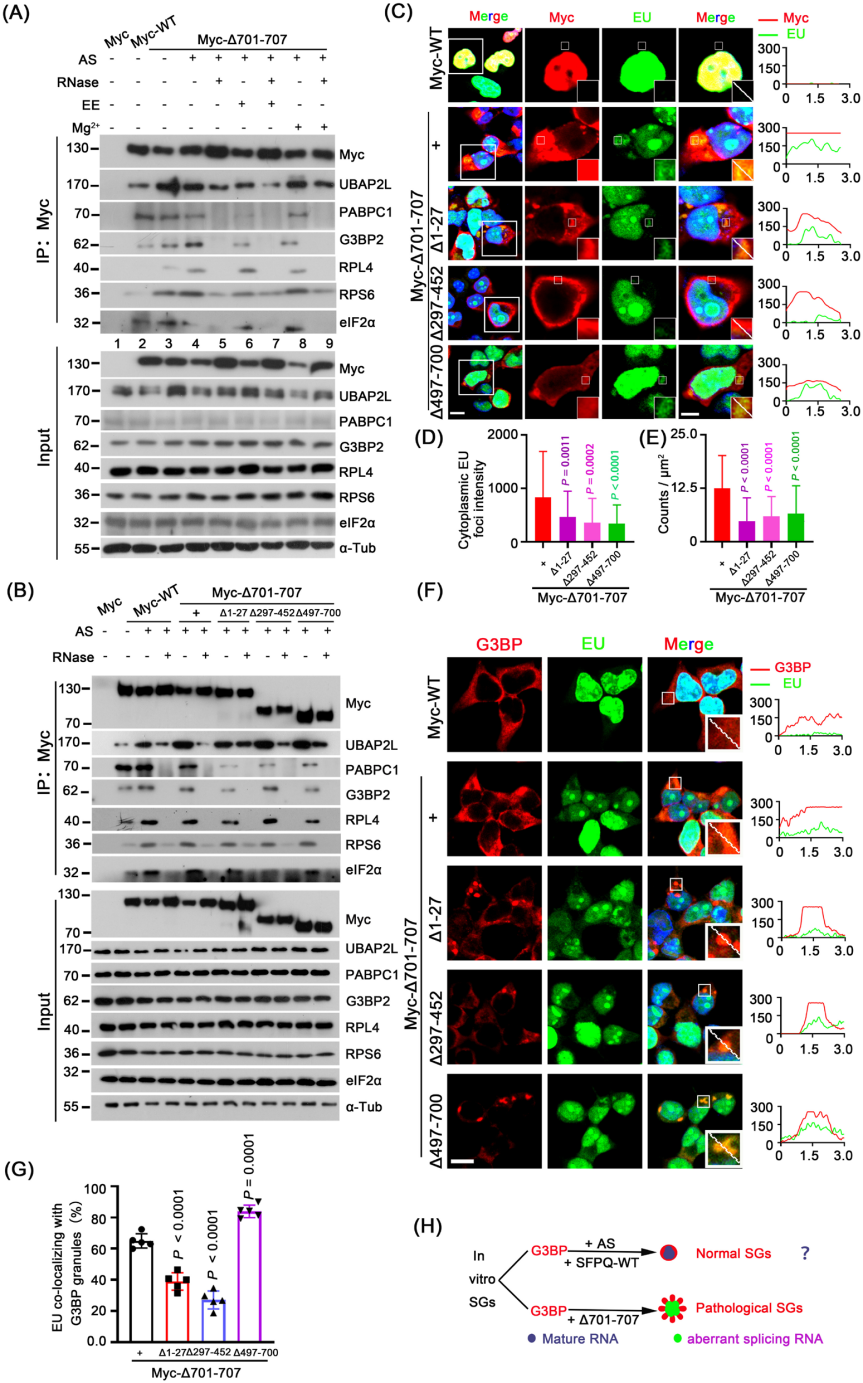
Ectopic cytoplasmic SFPQ leads to global RNAs aberrantly exported into cytoplasm where they associate with SGs. (A) Association of SFPQ△701‐707 with SG proteins by IP/WB. HEK293T cells expressing Myc‐SFPQ or Myc‐SFPQ△701‐707 were treated with/without AS (500 μM, 1 h), in combination with/without RNase A, EE (2 mM EDTA, 2.5 mM EGTA), or 5 mM MgCl_2_ in the lysis buffer. Proteins were precipitated using a Myc antibody. (B) Association of SFPQ deletants with SG proteins by IP/WB. HEK293T cells expressing SFPQ‐WT or indicated deletants were treated with/without AS (500 μM, 1 h), in combination with/without RNase A. (C–E) Distribution of newborn RNA (500 μM EU, 6 h, green) and its association with Myc (red) in HEK293T cells expressing Myc‐WT or indicated Myc‐tagged SFPQ deletants. Cells were incubated with EU for 48 h. Quantification of cytoplasmic EU intensity is shown in (D); focus size is expressed as fluorescence intensity in μm^2^ per focus and is shown in (E). Data are the mean ± standard deviation (SD) of *n* = 3 independent repeats. *P* values were determined using one‐way ANOVA with Tukey's multiple comparisons test, comparing Myc‐△701‐707 to other mutations. (F) Colocalisation of G3BP (red) and EU (green) in HEK293T cells, as treated in (C). (G) Percentages of cytoplasmic EU colocalised with G3BP granules in (F). Images are representative of 5 independent frames for each condition, and 20–35 granules were analysed per condition. Data are the mean ± standard deviation (SD) and represent three biologically independent experiments. *P* values were determined using one‐way ANOVA with Tukey's multiple comparisons test, comparing Myc‐△701‐707 to other mutations. (H) Schematic depicts that mislocalised SFPQ potentially lead to aberrant splicing RNAs being transported from nucleus to cytoplasm and associating with G3BP. All scale bars: 5 μm.

We next explored the mechanisms by which SFPQΔ701‐707 associates with SGs. We first tested whether SFPQΔ701‐707 interacts with SG components, including SG‐nucleating proteins (e.g., UBAP2L and G3BP2), RBPs (e.g., PABPC1), ribosomal subunits (e.g., ribosomal protein [RP] S6 and L4), and translation factors (e.g., eIF2α). Figure 3A and Figure S4A shows that (1) under normal conditions, SFPQ‐WT precipitated UBAP2L, G3BP2 and PABPC1 moderately, and RPS6, RPL4 and eIF2α weakly, while a significantly larger amount of these proteins was detected in the SFPQΔ701‐707 precipitates (compare lanes 2 and 3); (2) when stressed with AS, SFPQΔ701‐707 bound more RPS6 and RPL4, while its binding to SG components was RNA‐dependent, as the interactions were completely eliminated by RNase, except for UBAP2L and RPS6 (compare lanes 4 and 5); (3) ribosome‐dissociating EE decreased, while ribosome‐stabilising Mg^2+^ hardly increased the binding of SFPQΔ701‐707 to RBPs, indicating that an intact ribosome was necessary but not sufficient for recruiting RBPs (lanes 6–9); (4) RPS6 but not RPL4 was still present in the SFPQΔ701‐707 precipitates after RNase treatment, indicating that SFPQΔ701‐707 associated preferentially with the small 40S rather than the large 60S ribosomal subunit; and (5) the interaction between SFPQΔ701‐707 and UBAP2L was resistant to RNase, EE and Mg^2+^ (lanes 4–9), indicating a strong molecular interaction, which further supported the IP/WB data (Figure 1A–C). Taken together, these findings demonstrated that cytoplasmic SFPQ associates with RBPs and the 40S subunit protein, and simultaneously, via RNA, recruits more SG components to nucleate and promote SG assembly.

To further map the domains in SFPQΔ701‐707 responsible for these associations, IP revealed that in mutants devoid of each individual motif, SFPQΔ701‐707 association with SG components was greatly decreased but not abolished completely (Figure 3B, Figure S4B). This indicated that no one domain is solely responsible for recruiting SG components, but rather, all domains are required for the recruitment. In addition, each SFPQΔ701‐707 truncation precipitated UBAP2L and RPS6 even after RNase treatment (Figure 3B, Figure S4B), again corroborating their molecular interactions.

Finally, since aberrant mRNA export plays an important role in the formation of pathogenic SGs [9, 38], to examine if this also occurred for SFPQΔ701‐707, we used ethynyluridine (EU) to label newborn RNA [39] and then monitored global RNA export (Figure 3C–E). In the Myc‐WT group, the vast majority of EU was strictly limited within the nucleus. In sharp contrast, a considerable proportion of EU‐tagged transcripts were aberrantly exported from the nucleus and enclosed into cytoplasmic aggregates of SFPQ (Figure 3C–E). Loss of the RRM (Δ297‐452) most significantly compromised this export (Figure 3C,E). These observations suggest that cytoplasmic SFPQ induces the aberrant translocation of newborn RNA into the cytoplasm and SFPQ aggregates, and RRM is required for RNA incorporation. Meanwhile, the intensity and size of cytoplasmic RNA foci increased over time following the expression of cytoplasmic SFPQ, suggesting a spatiotemporal dependence on the accumulation of cytoplasmic SFPQ (Figure S4C‐F). Importantly, almost all of the cytoplasmic RNAs were uniformly colocalised with G3BP‐positive foci, and loss of the functional domains decreased the colocalisation (Figure 3F,G). As SFPQ is well‐established as a splicing factor [22], these results raise the possibility that mislocalised cytoplasmic SFPQ causes RNAs, which are controlled by its splicing, to be aberrantly transported into the cytoplasm, where they are incorporated into SFPQ aggregates and colocalise with SG proteins, forming abnormal RNA‐protein complexes that are prone to conversion into pathogenic SGs upon stress (Figure 3H).

### 3.5 Mislocalised Cytoplasmic SFPQ Impairs RNA Splicing and Disrupts Nucleocytoplasmic Transport in Cells

**FIGURE 4:**
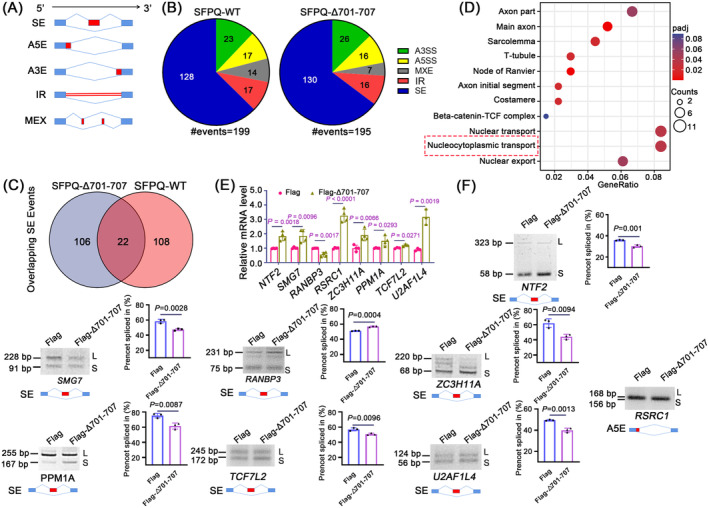
Cytoplasmic mislocation of SFPQ induces large changes in gene splicing and abnormal nucleocytoplasmic transport. (A) Schematics of alternative splicing events. SE, skipped exon; IR, intron retention; A5E, alternative 5′ splice site; A3E, alternative 3′ splice site; MXE, mutually exclusive exon. (B) Pie charts depicting distributions of significant splicing events in SFPQ‐WT and SFPQ△701‐707 after adjusting to control. (C) Venn diagram displaying overlap of SE events between SFPQ‐WT and SFPQ△701‐707 groups. (D) Dot graphs depicting GO terms associated with DEGs harbouring significant alternative splicing events in the SFPQ△701‐707 group. (E) qRT‐PCR quantifying the mRNA levels of indicated genes. Data are the mean ± standard deviation (SD). *n* = 4 for *NTF2*, *SMG7*, *RanBP3*, *RSRC1*, *ZC3H11A* and *PPM1A*; *n* = 3 for *TCF7L2* and *U2AF1L4*, unpaired Student's *t*‐test. (F) Electrophoresis validating specific alternative splicing for indicated genes. Schematic for gene‐specific alternative splicing is shown below the electrophoresis. l, long; s, short. Right panels showed the quantification of percent spliced in percent spliced index (PSI). Data are the mean ± standard deviation (SD) of *n* = 3 independent repeats and analysed by Student's t‐test.

To verify the above hypothesis, we next investigated whether and to what extent mislocalised SFPQ resulted in changes in alternative splicing events. We recorded a total of 199 and 195 significant splicing events in SH‐SY5Y cells expressing SFPQ‐WT and SFPQΔ701‐707 from the mRNA sequencing, using the Flag tag group as a baseline control (Figure 4A,B, Appendices S3 and S4). The proportions within the splicing patterns were comparable between the SFPQΔ701‐707 and SFPQ‐WT groups (Figure 4B), and for the special skipped exon (SE), which accounts for the largest proportion of total events, these two groups had relatively few target genes in common (Figure 4C). These data indicated that loss of the 701–707 aa NLS did not alter the splicing characteristics of SFPQ, but rather the target genes for splicing.

Intriguingly and importantly, GO analysis showed that DEGs with alternative splicing events in the SFPQΔ701‐707 group were primarily associated with nucleocytoplasmic transport (Figure 4D, Appendix S5). Nucleocytoplasmic transport factors have also been found to be components of SGs [40]. We therefore hypothesised that abnormal nucleocytoplasmic transport plays a critical role in the association between mislocalised cytoplasmic SFPQ and persistent SGs. To test this hypothesis, we first monitored the mRNA levels and alternative splicing of genes participating in the nucleocytoplasmic transport of RNAs and/or proteins (Figure 4E,F), namely, *NTF2*, *SMG7, RSRC1, ZC3H11A, TCF7L2, U2AF1L4*, *PPM1A* and *RanBP3*, which corroborated the changes revealed by the sequencing data (Appendix S5). This proved that mislocalised SFPQ causes aberrant splicing of nucleocytoplasmic transporters, disrupting nucleocytoplasmic transport in cells.

**FIGURE 5:**
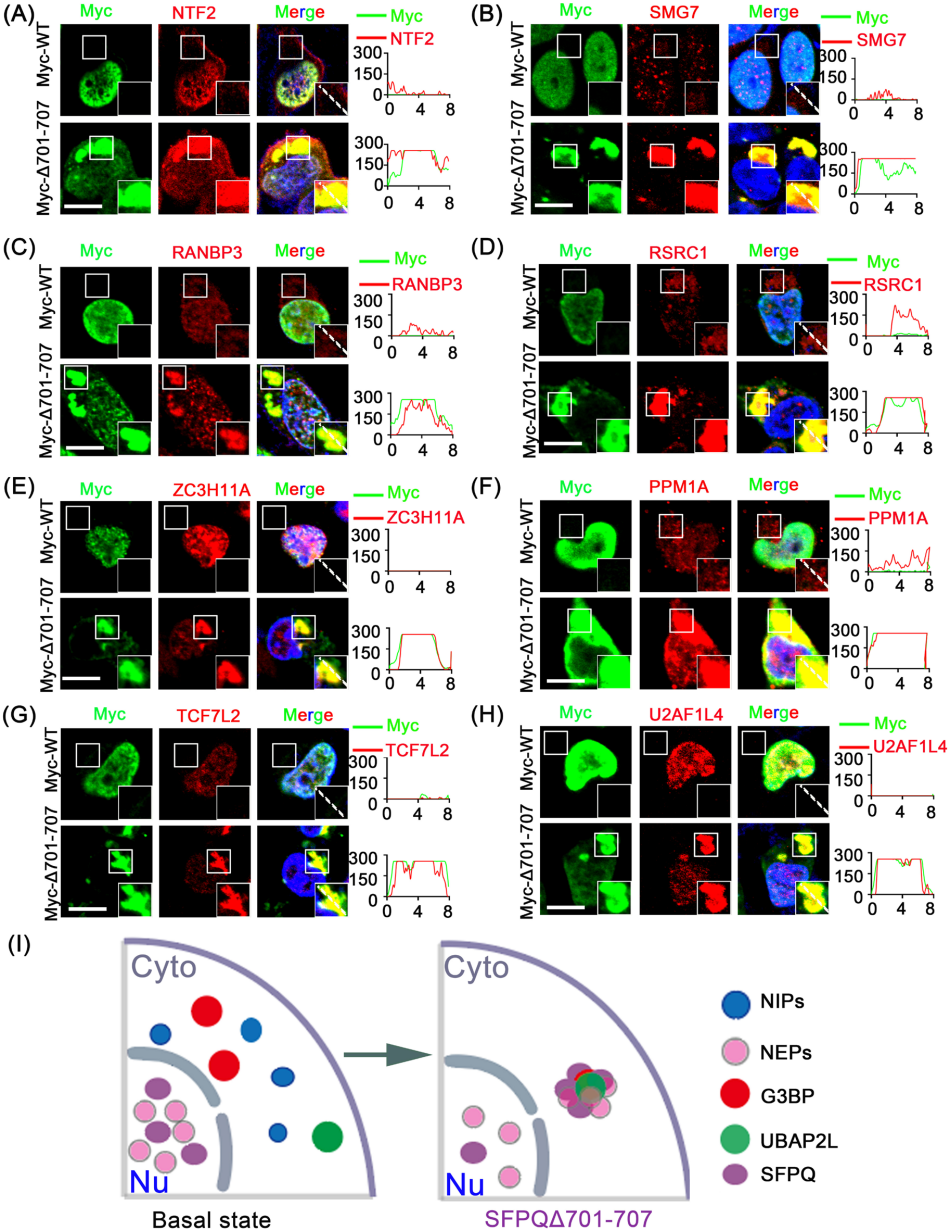
Mislocalised cytoplasmic SFPQ causes aberrant translocation of nucleocytoplasmic transporters to the cytoplasm and aggregation with SFPQ deletants. (A–H) Representative images of the association of nucleocytoplasmic transporters with Myc in HEK293T cells expressing Myc‐WT or Myc‐△701‐707. Line scans show the intensity profiles of Myc along with indicated nucleocytoplasmic transporters. (I) Schematic depicts that mislocalised SFPQ aggregates with nucleocytoplasmic transporters and SG proteins, forming aberrant protein complexes. Cyto, cytoplasm; NEPs, nuclear export proteins; NIPs, nuclear import proteins; Nu, nucleus. All scale bars: 10 μm.

To confirm the contribution of dysfunctional nucleocytoplasmic transporters to pathological SGs, we first tested whether these transporters are involved in SGs. We stressed HEK293T cells with AS or NaCl and detected the colocalisation of the transporters with G3BP by IF. As shown in Figure S5, NTF2, SMG7 and RanBP3 clearly, RSRC1 and ZC3H11A moderately and PPM1A, TCF7L2 and U2AF1L4 sparsely colocalised with G3BP granules upon stress, indicating that these transporters are also involved in SGs. In combination with the observation that these transporters underwent differential splicing, we asked whether mislocalised cytoplasmic SFPQ causes aberrant nucleocytoplasmic distribution of these transporters and incorporation into SFPQ aggregates. HEK293T cells expressing Myc‐SFPQ‐WT or indicating SFPQ deletants without stress were tested. As shown in Figure 5A–H and Figure S6A–H, all tested transporters were notably included into SFPQΔ701‐707 aggregates, and loss of the RRM, but not others, appeared to be associated with a reduced likelihood of inclusion into these aggregates. Nevertheless, no association with SFPQ‐WT was observed. SFPQ deletants showed mild effects on the protein levels of the transporters (Figure S6I, J). Importantly, when examining their association with SG proteins, we found that all cytoplasmic counterparts of these transporters were significantly colocalised with G3BP and formed clear granules without stress (Figure S7F–L). In combination with the above findings, our data cumulatively demonstrated that mislocalised cytoplasmic SFPQ causes aberrant splicing of nucleocytoplasmic transporters. Consequently, these transporters aberrantly accumulated in SFPQ aggregates and colocalised with G3BP granules, forming abnormal protein aggregates (Figure 5I).

### 3.6 Blockage of Nuclear Export or Splicing Does Alleviate Aberrant Cytoplasmic RNA–Protein Aggregates

Finally, we aimed to determine the contributions of SG formation, nucleocytoplasmic transport, and alternative splicing to aberrant cytoplasmic aggregates. We used three approaches: (1) inhibition of SG nucleation by knocking down G3BP1 and G3BP2 [32, 40, 41], (2) selective inhibition of nuclear export by KPT‐330 [41] and (3) inhibition of alternative splicing by Pladienolide B (PlaB) [39] (Figure S7A). We first examined the contribution of SGs, because SG assembly impairs nucleocytoplasmic transport. G3BP knockdown does not assemble SGs when treated with AS [19, 40, 41]. HEK293T cells expressing siRNAs targeting *G3BP1* and *2* were transfected with SFPQΔ701‐707 (Figure S7B). Both G3BP1 and 2 expressions were almost cleared (Figure S7C,D). However, *G3BP1*/*2* knockdown did not block the cytoplasmic accumulation of SFPQΔ701‐707 (Figure S7E). Similarly, *G3BP1*/*2* knockdown hardly prevented the transporters from being translocated into the cytoplasm and colocalising with G3BP granules induced by SFPQΔ701‐707, although the size of NTF2, SMG7, RSRC1 and PPM1A granules were decreased (Figure S7F–M). These data exclude SGs as the critical cause of the cytoplasmic aggregations of SFPQ and transporters.

**FIGURE 6:**
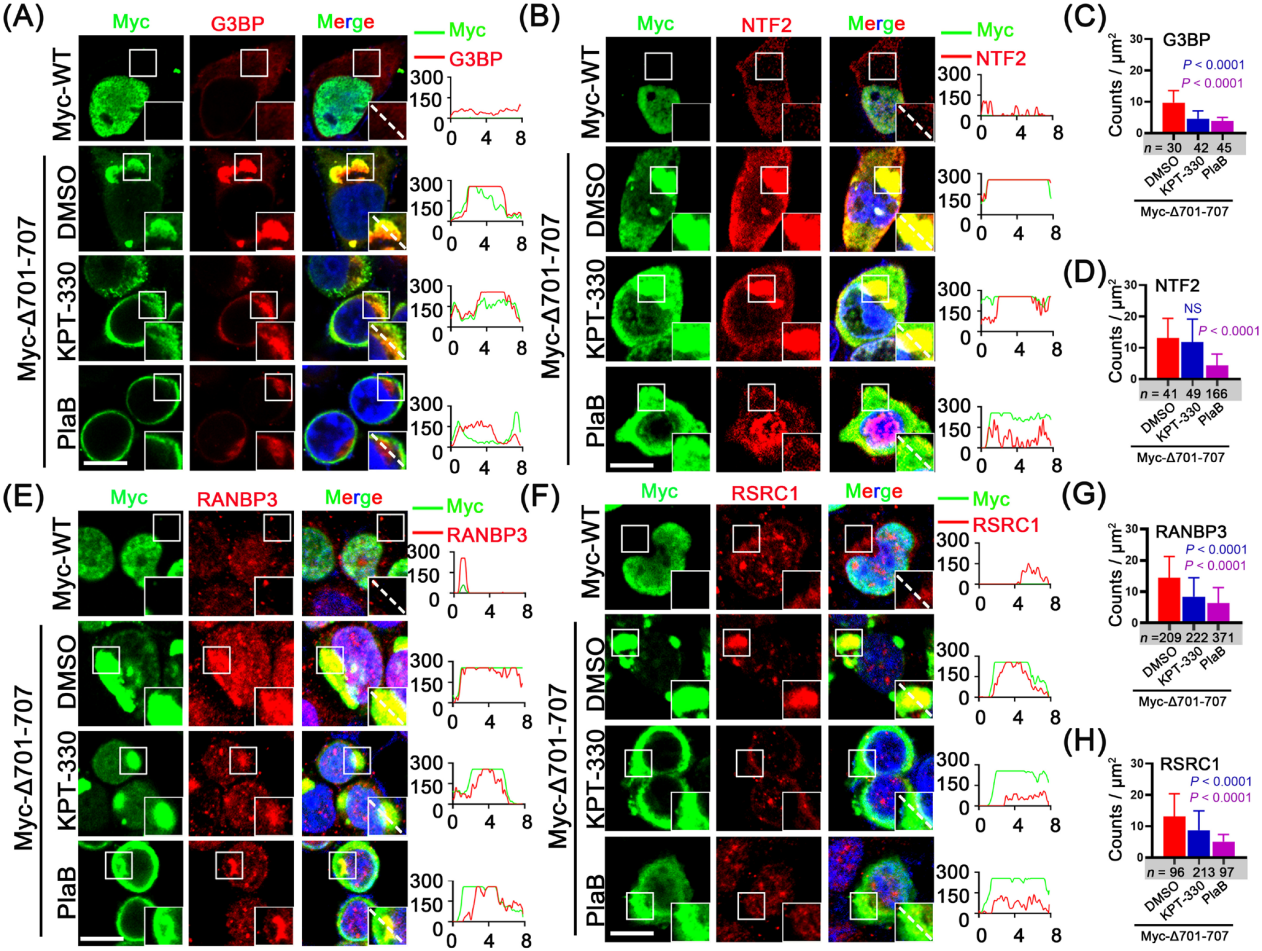
KPT‐330 and Plab moderately alleviate the sequestration of nucleocytoplasmic transporters into cytoplasmic SFPQ aggregates. (A, C) HEK293T cells expressing Myc‐WT or Myc‐△701‐707 were treated with DMSO, KPT‐330 (3 μM, 24 h) or Plab (10 μM, 6 h), followed by IF of Myc and G3BP. (C) Quantification is expressed of G3BP as the fluorescence intensity in μm^2^ per focus in (A). (B, D) HEK293T cells expressing Myc‐WT or Myc‐△701‐707 were treated with DMSO, KPT‐330 (3 μM, 24 h) or Plab (10 μM, 6 h), followed by IF of Myc and NTF2. (D) Quantification is expressed of NTF2 as the fluorescence intensity in μm^2^ per focus in (B). (E, G) HEK293T cells expressing Myc‐WT or Myc‐△701‐707 were treated with DMSO, KPT‐330 (3 μM, 24 h) or Plab (10 μM, 6 h), followed by IF of Myc and RANBP3. (G) Quantification is expressed of RANBP3 as the fluorescence intensity in μm^2^ per focus in (E). (F, H) HEK293T cells expressing Myc‐WT or Myc‐△701‐707 were treated with DMSO, KPT‐330 (3 μM, 24 h) or Plab (10 μM, 6 h), followed by IF of Myc and RSRC1. (H) Quantification is expressed of RSRC1 as the fluorescence intensity in μm^2^ per focus in (F). All scale bars: 10 μm. Data are the mean ± standard deviation (SD) of *n* = 3 independent repeats. Student's *t*‐test (*p* > 0.05).

We next tested the contributions of nuclear exporters. A previous study revealed that the primary nuclear exporter chromosome region maintenance 1 protein homologue (CRM1)/exportin‐1 (XPO1), one of the seven exportins, mediated the transport of over 220 proteins and numerous mRNAs [42]. Meanwhile, Ran‐binding protein 3 (RanBP3) is a cofactor for CRM1‐mediated nuclear protein export [43], and protein phosphatase 1A (PPM1A) can dephosphorylate RanBP3 to enable efficient nuclear export [44]. Therefore, we tested whether inhibition of CRM1‐mediated nuclear export by KPT‐330 could prevent the cytoplasmic aggregation (Figure S7A,B). Firstly, in the SFPQ‐WT group, KPT‐330 treatment did not induce colocalisation of Myc with G3BP (Figure S8B). In contrast, in SFPQΔ701‐707 cells, KPT‐330 treatment significantly decreased the cytoplasmic accumulation of G3BP compared with DMSO treatment (Figure 6A,C). Additionally, KPT‐330 treatment reduced the cytoplasmic accumulation of the transporters RANBP3, PPM1A, SMG7, ZC3H11A, TCF7L2 and U2AF1L4, which mediate nuclear export (Figure 6E,G, Figure S8C‐L). Moreover, RSRC1, an alternative 5′ splicing protein, also exhibited reduced cytoplasmic accumulation after KPT‐330 treatment (Figure 6F,H). Correspondingly, their association with cytoplasmic SFPQ was significantly decreased. However, KPT‐330 treatment did not affect nuclear transport factor 2 (NTF2; Figure 6B,D), which mediates nuclear import, further confirming the efficacy and specificity of KPT‐330 treatment. Of note, KPT‐330 treatment caused SFPQ to disperse in the cytoplasm (Figure 6A–H, Figure S8C–L); as a result, both SFPQ aggregation and G3BP granules were largely diminished (Figure 6A,C). These data demonstrated that disorders in nucleocytoplasmic transport are the critical triggers for cytoplasmic aggregation of SFPQ, transporters and SG nucleators.

Our sequencing data revealed a substantial number of alternative splicing events. Based on this, we then evaluated the effect of the splicing inhibitor, PlaB (Figure S7A,B). The abnormal splicing events were also replicated in HEK293T cells, and PlaB treatment effectively restored normal splicing to nearly control levels (Myc‐tag group, Figure S8A). PlaB treatment did not induce the colocalisation of Myc with G3BP in the SFPQ‐WT group (Figure S8B), and most importantly, avoided incorporation of transporters and G3BP granules into cytoplasmic SFPQ aggregates compared to DMSO treatment in the SFPQΔ701–707 group (Figure 6A–H, Figure S8C‐L). Thus far, we can conclude that mislocalised SFPQ‐induced aberrant splicing is predominantly responsible for the cytoplasmic aggregates of transporters and SG proteins.

**FIGURE 7:**
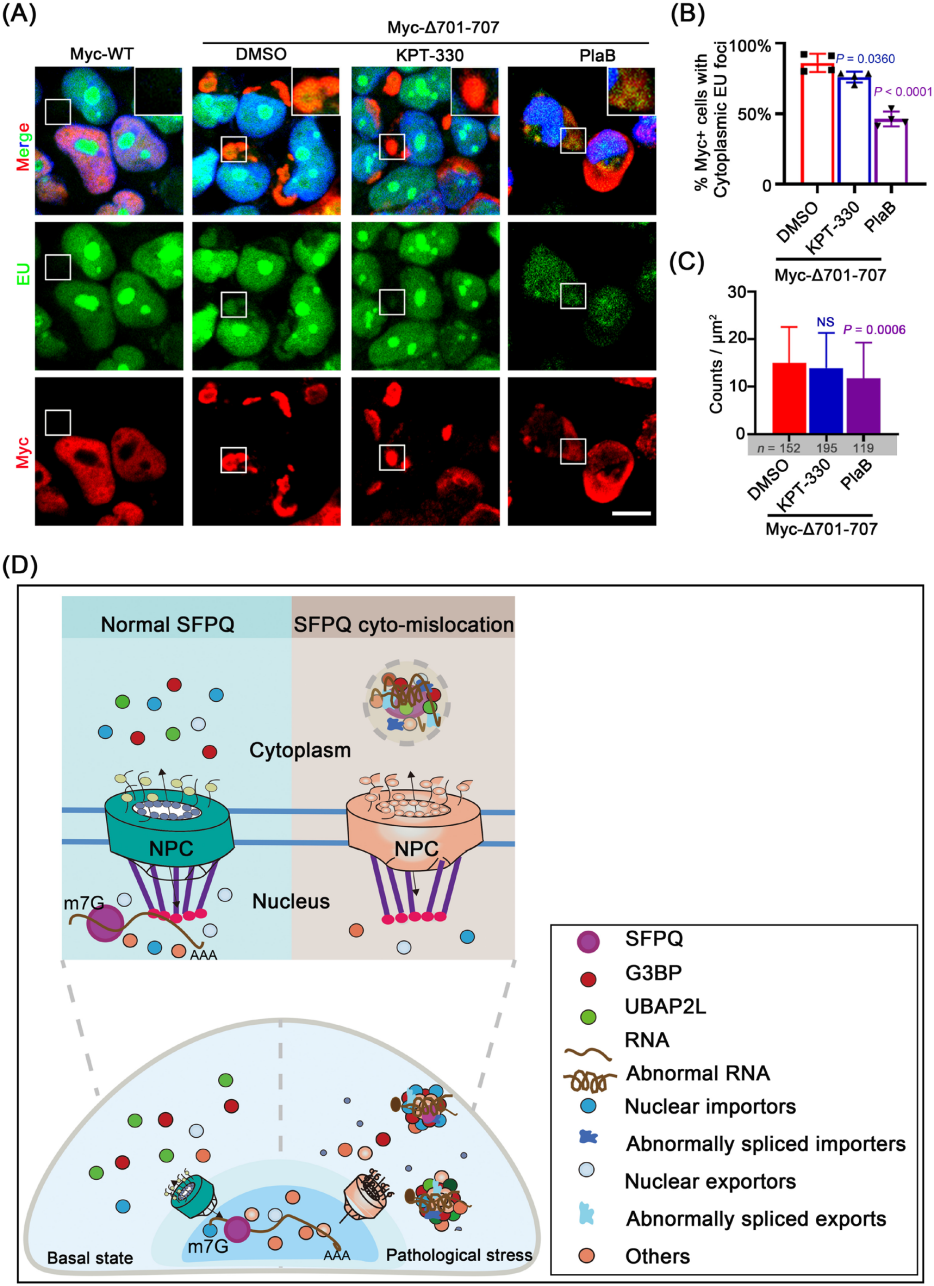
KPT‐330 and Plab mitigate the sequestration of global RNAs into cytoplasmic SFPQ aggregates. (A–C) Representative images of the distribution of RNA (EU, green) in HEK293T cells expressing Myc‐WT or Myc‐△701‐707 after treatment with DMSO, KPT‐330 (3 μM, 24 h) or Plab (10 μM, 6 h). Quantification is expressed as the percentage of Myc‐positive cells with cytoplasmic EU foci and is shown in (B). Data are the mean ± standard deviation (SD) of *n* = 4 independent repeats, student's *t*‐test. (C) Focus size is expressed as fluorescence intensity in μm^2^ per focus. Data are the mean ± standard deviation (SD), student's t‐test. NS, not significant (*p* > 0.05). Scale bars: 10 μm. (D) Working model depicts how mislocalised cytoplasmic SFPQ results in pathogenic SGs. In normal cells, SFPQ shuttles between nucleus and cytoplasm for correct transcriptional regulation, RNA processing and cytoplasmic trafficking. In the pathological state, SFPQ is mislocalised into the cytoplasm and forms aggregates. This leads to aberrant splicing of nucleocytoplasmic transporters and accordingly disruption in nucleocytoplasmic transport. As a result, nucleocytoplasmic transporters and abnormal RNAs are sequestered into SFPQ aggregates, concurrent with stubborn SGs forming very aberrant RNA‐protein complexes, leading to the pathogenic SG formation.

The accumulation of export factors in SGs is not necessarily the direct cause of the impairment of mRNA export, since inhibiting the formation of SGs did not release the obstruction [39]. We therefore tested whether KPT‐330 or PlaB treatment could also block abnormal mRNA export induced by SFPQ mislocalisation. We observed that both KPT‐330 and PlaB reversed the RNA export induced by SFPQΔ701‐707 in HEK293T cells, with PlaB exhibiting a stronger effect, thereby avoiding RNA inclusion into SFPQ aggregates (Figure 7A–C).

## 4 Discussion

SGs are dynamic structures that rapidly form and disperse with acute stress, but chronic illness and/or persistent stress induces SGs to form more stable complexes, disrupting RNA metabolism and protein translation [9]. Here, we investigated the cellular roles of mislocalised cytoplasmic SFPQ in the regulation of SG dynamics, RNA metabolism and the underlying molecular mechanisms. We summarise our key findings as follows: (1) endogenous SFPQ was predominantly localised in the nucleus and sparsely translocated to the cytoplasm or colocalised with SGs under acute stress; (2) exogenous cytoplasmic SFPQ spontaneously formed cytoplasmic aggregates, which associated with SG proteins under physiological conditions, thereby disturbing SG dynamics; (3) the RMM was a prerequisite for SFPQ aggregates to recruit RNA and RBPs to promote SG assembly; (4) mislocalised SFPQ induced a profound change in the splicing of genes, with a subset of transcripts highly associated with nucleocytoplasmic transport and axon development, causing RNA and transporters trapped into cytoplasmic SFPQ aggregates to colocalise with SG proteins to form very aberrant RNA‐protein complexes; and finally (5) inhibition of nuclear export or alternative splicing, but not SG nucleation, attenuated aberrant RNA‐protein aggregates induced by mislocalised SFPQ. Based on these findings, we propose a model in which under physiological conditions, SFPQ is balanced between nuclear and cytoplasmic localisation to maintain proper gene expression and RNA processing. Under pathological conditions (e.g., gene mutation and chronic stress), SFPQ is aberrantly translocated into the cytoplasm and induces abnormal splicing of nucleocytoplasmic transporters, thereby disrupting nucleocytoplasmic transport and resulting in transporters and RNAs being further sequestered into SFPQ aggregates. These aggregates then incorporate persistent SGs to form aberrant RNA–protein complexes, which disrupt RNA metabolism and lead to cell injury (Figure 7D).

This study provides new insights into the functional and molecular correlations among RBPs, SGs and nucleocytoplasmic transport. First, we propose that the coalescence of SFPQ aggregates with SGs is more likely a causative factor than an outcome. Evidence supporting this notion comes from the observation that SFPQΔ701‐707 directly gathered SG components and formed physiological SGs, even without stress, leading to persistent SGs. Moreover, disruption of SG nucleation by knocking down *G3BP1/2* could not rescue aberrantly spliced transporters and abnormal RNAs from being incorporated into SFPQ aggregates. Second, we determined whether the loss of normal nuclear function or gain of toxic cytoplasmic function induces neuronal damage [45]. Such a debate has been ongoing since the deletion of RBPs, such as TDP‐43, from the nucleus and their accumulation in the cytoplasm was discovered in degenerating neurons. We suggest that the gain of toxic function by SFPQ in the cytoplasm, beyond the loss of normal nuclear function, is the bona fide trigger for the observed changes. Besides persistent SGs, mislocalised SFPQ also caused RNA and transporters to become further enclosed into aberrant cytoplasmic aggregates to form the foundation of persistent SGs. Hence, we propose that, in this case, mislocalised SFPQ uses itself as a template in the cytoplasm to form preinclusions, which act as a ‘core’, according to the ‘core first’ model for SG assembly [4], to recruit more SG components to form larger aggregates upon stress. Further exacerbated by the excessive self‐assembly activity of SFPQ, RNA or RBPs are trapped in these pathological inclusions and cannot be released into the cytoplasm even after stress resolves. Based on these findings, we conclude that all cellular phenotypes can be exclusively attributed to the cytoplasmic mislocalisation of SFPQ.

An equally important question that remains unresolved is the identity of the factor that induces cytoplasmic mislocalisation of SFPQ, especially in patients. The first possibility is that there may be potential mutation(s) located in the NLS in SFPQ, including the aa 547–754 NLS, as it is also necessary for the correct nuclear location of SFPQ, particularly in SH‐SY5Y cells. Alternatively, there may be potential aberrant post‐translational events (e.g., phosphorylation [46, 47] within the NLS or methylation events within the RGG box [48]), leading to abnormal localisation. Second, cofactors that mediate SFPQ nuclear export must exist, because unlike FUS [49], SFPQ has no nuclear export sequence [22, 23]; however, such cofactors have not yet been identified. Any abnormality in the expression or function of such a cofactor would likely lead to the cytoplasmic mislocalisation of SFPQ. Finally, the NONA/ParaSpeckle [22, 23] and coiled‐coiled domains [24] are required for both SFPQ nuclear localisation and complex formation with NONO/PSPC1. It is therefore probable that NONO, PSPC1 or other potential nuclear partners mediate the nuclear retention of SFPQ and simultaneously coordinate with the nuclear exporter to maintain the balance between its nuclear and cytoplasmic localisation, particularly in neurons. Evidence supporting this notion comes from the finding that mutations identified in the coiled‐coil domain lead to abnormal localisation [24].

In summary, we elucidated the cytopathological influences and mechanisms underlying cytoplasmic mislocalisation of SFPQ in nucleocytoplasmic transport and pathological SGs. Furthermore, we highlighted the integrative roles of SFPQ in RNA transcription and processing and in nucleocytoplasmic transport. The profound changes in gene splicing products disrupt nucleocytoplasmic transport, which subsequently sequesters critical RNAs and transport factors into cytoplasmic inclusions, constituting the etiological correlation between cytoplasmic mislocalisation of SFPQ and pathologically persistent SGs. Future research is required to determine the exact cause of cytoplasmic mislocalisation of SFPQ and the suppression of aberrant SG aggregates. Finally, the recognition that cytoplasmic mislocalisation has occurred, disconnecting SGs from SFPQ inclusions, or blocking efforts to release RNAs and RBPs into the cytoplasm may have implications for treating or attenuating neuronal failures. Our data suggest the possible therapeutic application of inhibitors of nucleocytoplasmic transport or alternative splicing to treat SFPQ‐associated pathologies.

## Author Contributions

**Zicong Huang:** conceptualization (lead), data curation (lead), formal analysis (lead), investigation (lead), methodology (lead), project administration (lead), resources (lead), supervision (lead), validation (lead), writing – original draft (lead), writing – review and editing (lead). **Hanbin Zhang:** formal analysis (equal), investigation (equal), methodology (equal), project administration (equal), resources (equal), supervision (equal), writing – original draft (equal), writing – review and editing (equal). **Chuyu Huang:** conceptualization (lead), formal analysis (equal), methodology (equal), project administration (equal), resources (lead), supervision (equal), validation (equal), visualization (equal), writing – original draft (equal). **Runduan Yi:** conceptualization (equal), data curation (equal), formal analysis (equal), writing – original draft (lead), writing – review and editing (equal). **Xiaoyuan Zhang:** formal analysis (equal), investigation (equal), methodology (equal). **Ke Ma:** formal analysis (equal), investigation (equal), methodology (equal). **Wei Huang:** methodology (equal), resources (equal), visualization (equal). **Qingqing Wu:** formal analysis (equal), methodology (equal), resources (equal). **Yuge Zhuang:** formal analysis (equal), investigation (equal), methodology (equal). **Jinsheng Liu:** methodology (equal), resources (equal), validation (equal). **Wenyuan Liu:** methodology (equal), resources (equal), validation (equal). **Yunhui Guo:** methodology (equal), resources (equal), validation (equal). **Xiangjin Kang:** formal analysis (equal), supervision (equal), writing – original draft (equal), writing – review and editing (equal). **Xiao Hu:** formal analysis (equal), supervision (equal), writing – review and editing (equal). **Xiaochun Bai:** conceptualization (equal), formal analysis (equal), project administration (equal), supervision (equal), writing – review and editing (equal). **Zhenguo Chen:** conceptualization (equal), data curation (equal), formal analysis (lead), funding acquisition (lead), investigation (equal), methodology (equal), project administration (equal), resources (lead), supervision (equal), validation (equal), visualization (equal), writing – original draft (lead), writing – review and editing (lead).

## Conflicts of Interest

The authors declare no conflicts of interest.

## Supporting information


**Appendix S1** DEGs in SH‐SY5Y Cells Transfected with Flag‐SFPQ‐WT or Flag‐SFPQ△701‐707 versus Flag Tag.xlsx (v1.0).

**Appendix S2** KEGG Pathway Analysis of Upregulated DEGs in Flag‐SFPQ△701‐707 Group versus Flag Tag.xlsx (v1.0).

**Appendix S3** DEGs with Significant Alternative Splicing Events in SH‐SY5Y Cells Transfected with Flag‐SFPQ‐WT versus Flag Tag.xlsx (v1.0).

**Appendix S4** DEGs with Significant Alternative Splicing Events in SH‐SY5Y Cells Transfected with Flag‐SFPQ△701‐707 versus Flag Tag.xlsx (v1.0).

**Appendix S5** GO Analysis of DEGs with Alternative Splicing Events in Flag‐SFPQ△701‐707 Group versus Flag Tag.xlsx (v1.0).

Appendix S6

## Data Availability

RNA‐seq data have been deposited in the NCBI database (PRJNA886326). Other data are available upon reasonable request.
