# Supplementary material for: Cytoplasmic Aggregates of Splicing Factor Proline‐Glutamine Rich Disrupt Nucleocytoplasmic Transport and Induce Persistent Stress Granules
Source: J Cell Mol Med. 2024 Dec 5;28(23):e70261. doi: 10.1111/jcmm.70261 (PMC11619156; doi:10.1111/jcmm.70261)
Supplement: Supplementary file 6 — Appendix S6 [file JCMM-28-e70261-s004.pdf]

Supplemental Information for

Cytoplasmic aggregates of splicing factor proline-glutamine rich disrupt nucleocytoplasmic transport and induce persistent stress granules

Zicong Huang, Hanbin Zhang, Chuyu Huang, Runduan Yi, Xiaoyuan Zhang, Ke Ma, Wei Huang, Qingqing Wu, Yuge Zhuang, Jinsheng Liu, Wenyuan Liu, Yunhui Guo, Xiangjin Kang, Xiao Hu, Xiaochun Bai, Zhenguo Chen

Correspondence: Zhenguo Chen, e-mail: czg1984@smu.edu.cn.

This PDF file includes:

Supplementary Methods

Supplementary Text

8 Supplementary Figures

2 Supplementary Tables

Supplementary Methods

SDS-PAGE and in-gel digestion

Immunoprecipitates were eluted from the beads by incubation with SDS loading buffer in boiling water bath for 10 min and then separated by SDS-PAGE. For in-gel digestion, gels were first visualized using a silver staining kit (Thermo Fisher Scientific, Waltham, MA, USA), and then targeted gel lanes were picked and digested with trypsin.

High-pH reversed-phase chromatography

The Ultimate 3000 HPLC system (Dionex, USA) equipped with a 2.00-mm-inner diameter *100-mm-long Gemini-NX 3u C18110Acolumns (Phenomenex, USA) was used for High-pH fractionation. Peptides were loaded onto the column and washed isocratically at 95% eluent A (20 mM $HCOONH_4$, 2M NaOH) (pH10). The tryptic peptides fractionation was performed using a linear binary gradient from 15% to 50% B (20 mM $HCOONH_4$, 2 M NaOH, 80% ACN) (pH 10) at 0.2ml/min for more than 45 min. Finally, the column was washed at 90% B for 10 min and returned to 95% A for 10 min. Set the UV detector was at 214/280 nm, and fractions were collected every 1 min. 10 fractions were pooled and dried by vacuum centrifuge for subsequent nano-reversed phase liquid chromatography (nano-LC) fractionation.

RPLC-MS/MS analysis

The fraction was resuspended in loading buffer (0.1% FA,2% ACN) and separated with an Ultimate 3000 nano-LC system equipped with a C18 reverse phase column (100-μm inner diameter, 10-cm long, 3-μm resin from MichromBioresources, Auburn, CA). Separate the peptides with the following parameters: 1) mobile phase A:0.1% FA, 5% ACN, dissolved in water; 2) mobile phase B: 0.1% FA, 95% ACN; 3) flow rate: 300nl/min; 4) gradient: B-phase increased from 5% to 40%,70min. Then, the LC eluent was subject to Q Exactive (Thermo Fisher) in an information dependent acquisition mode. MS spectra were acquired across the mass range of 400-1,250 m/z in high resolution mode (> 30, 000) using 250 ms accumulation time per spectrum. A maximum of 20 precursors per cycle were chosen for fragmentation from each MS spectrum with 100 ms minimum accumulation time for each precursor and dynamic

exclusion for 20 s. Tandem mass spectra were recorded in high sensitivity mode (resolution > 15,000) with rolling collision energy on. Raw data were searched against the Uniprot human protein database using the SEQUEST algorithm embedded in the Protein Discoverer 1.3 Software (Thermo Fisher Scientific, Waltham, MA, USA). The following parameters were applied during the database search: 10 ppm precursor mass error tolerance, 1 Da fragment mass error tolerance, static modifications of carbamidomethylation for all cysteine residues, flexible modification of oxidation modifications for methionine residues, and one missed cleavage site of trypsin was allowed. FDR <0.01 was used as filtering criteria for all identified peptides. Only proteins identified with two or more unique peptides were considered, and proteins identified with the same set of peptides were grouped.

RNA purification and library preparation for transcriptome sequencing

Total RNA was purified using the Trizol reagent (Invitrogen; Carlsbad, CA, USA). RNA integrity was assessed using the RNA Nano 6000 Assay Kit of the Bioanalyzer 2100 system (Agilent Technologies, CA, USA). mRNA was purified from total RNA using poly-T oligo-attached magnetic beads. Fragmentation was carried out using divalent cations under elevated temperature in First Strand Synthesis Reaction Buffer (5X). First strand cDNA was synthesized using random hexamer primer and M-MuLV Reverse Transcriptase, then use RNaseH to degrade the RNA. Second strand cDNA synthesis was subsequently performed using DNA Polymerase I and dNTP. Remaining overhangs were converted into blunt ends via exonuclease/polymerase activities. After adenylation of 3’ ends of DNA fragments, Adaptor with hairpin loop structure were ligated to prepare for hybridization. In order to select cDNA fragments of preferentially 370~420 bp in length, the library fragments were purified with AMPure XP system (Beckman Coulter, Beverly, USA). Then PCR was performed with Phusion High-Fidelity DNA polymerase, Universal PCR primers and Index (X) Primer. At last, PCR products were purified (AMPure XP system) and library quality was assessed on the Agilent Bioanalyzer 2100 system.

Clustering and sequencing

The clustering of the index-coded samples was performed on a cBot Cluster

Generation System using TruSeq PE Cluster Kit v3-cBot-HS (Illumia) according to the manufacturer's instructions. After cluster generation, the library preparations were sequenced on an Illumina Novaseq platform and 150 bp paired-end reads were generated.

Data Analysis

Quality control

Raw data (raw reads) of fastq format were firstly processed through in-house perl scripts. In this step, clean data (clean reads) were obtained by removing reads containing adapter, reads containing N base and low quality reads from raw data. At the same time, Q20, Q30 and GC content the clean data were calculated. All the downstream analyses were based on the clean data with high quality.

Reads mapping to the reference genome

Reference genome and gene model annotation files were downloaded from genome website directly. Index of the reference genome was built using Hisat2 v2.0.5 and paired-end clean reads were aligned to the reference genome using Hisat2 v2.0.5. We selected Hisat2 as the mapping tool for that Hisat2 can generate a database of splice junctions based on the gene model annotation file and thus a better mapping result than other non-splice mapping tools.

Quantification of gene expression level

Feature Counts v1.5.0-p3 was used to count the reads numbers mapped to each gene. And then FPKM of each gene was calculated based on the length of the gene and reads count mapped to this gene. FPKM, expected number of Fragments Per Kilobase of transcript sequence per Millions base pairs sequenced, considers the effect of sequencing depth and gene length for the reads count at the same time, and is currently the most commonly used method for estimating gene expression levels.

Differential expression analysis

Differential expression analysis of two conditions/groups (two biological replicates per condition) was performed using the DESeq2 R package (1.20.0). DESeq2 provide statistical routines for determining differential expression in digital gene expression data using a model based on the negative binomial distribution. The resulting

P-values were adjusted using the Benjamini and Hochberg's approach for controlling the false discovery rate. Genes with an adjusted *P*-value <0.05 found by DESeq2 were assigned as differentially expressed.

threshold for significantly differential expression.

Enrichment analysis of differentially expressed genes (DEGs)

Gene Ontology (GO) enrichment analysis of DEGs was implemented by the clusterProfiler R package, in which gene length bias was corrected. GO terms with corrected *P* value less than 0.05 were considered significantly enriched by DEGs. Kyoto Encyclopedia of Genes and Genomes (KEGG) is a database resource for understanding high-level functions and utilities of the biological system, such as the cell, the organism and the ecosystem, from molecular-level information, especially large-scale molecular datasets generated by genome sequencing and other high-through put experimental technologies (http://www.genome.jp/kegg/). We used clusterProfiler R package to test the statistical enrichment of differential expression genes in KEGG pathways. The Reactome database brings together the various reactions and biological pathways of human model species. Reactome pathways with corrected Pvalue less than 0.05 were considered significantly enriched by differential expressed genes. The DO (Disease Ontology) database describes the function of human genes and diseases. DO pathways with corrected *P* value less than 0.05 were considered significantly enriched by differential expressed genes. The DisGeNET database integrates human disease-related genes. DisGeNET pathways with corrected *P* value less than 0.05 were considered significantly enriched by differential expressed genes. We used clusterProfiler software to test the statistical enrichment of DEGs in the Reactome pathway, the DO pathway, and the DisGeNET pathway.

Gene Set Enrichment Analysis (GSEA)

GSEA is a computational approach to determine if a pre-defined Gene Set can show a significant consistent difference between two biological states. The genes were ranked according to the degree of differential expression in the two samples, and then the predefined Gene Set were tested to see if they were enriched at the top or bottom of the list. Gene set enrichment analysis can include subtle expression changes.

We use the local version of the GSEA analysis tool http://www.broadinstitute.org/gsea/index.jsp, GO, KEGG, Reactome, DO and DisGeNET data sets were used for GSEA independently.

Single Nucleotide Polymorphism (SNP) analysis

GATK2 (v3.7) software was used to perform SNP calling. Raw vcf files were filtered with GATK standard filter method and other parameters (cluster:3; WindowSize:35; QD < 2.0 o; FS > 30.0; DP < 10.

Alternative Splicing (AS) analysis

AS is an important mechanism for regulate the expression of genes and the variable of protein. rMATS(3.2.5) software was used to analysis the AS event.

Protein-Protein Interactions (PPI) analysis of DEGs

PPI analysis of DEGs was based on the STRING database, which known and predicted Protein-Protein Interactions.

Fusion analysis

Fusion gene refers to the chimeric gene formed by the fusion of all or part of the sequences of two genes, which is generally caused by chromosome translocation, deletion and other reasons. We used Starfusion software (1.2.0) to detect genes that are fused. Star-fusion is a software package uses fusion output results of STAR alignment to detect fusion transcripts, including SATR alignment, SATRfusion. predict, SATR-fusion.filter was used to correct the predicted results of Star-fusion to ensure the accuracy of the results.

Supplementary Text

Nominal mass (M_r): **76255**; Calculated pI value: **9.50**
NCBI BLAST search of gi|29881667 against nr
Unformatted sequence string for pasting into other applications
Taxonomy: Homo sapiens
Fixed modifications: Carbamidomethyl (C)
Variable modifications: Oxidation (M)
Cleavage by Trypsin: cuts C-term side of KR unless next residue is P
Sequence Coverage: **13%**

Matched peptides shown in **Bold Red**

1 MSRDRFRSRG GGGGGFHRRG GGGGRGGLHD FR**SPPPGMGL NQNR**GPMGPG
51 PGQSGPKPPI PPPPPHQQQQ QPPPQQPPPQ QPPPHQPPPH PQPHQQQQPP
101 PPPQDSSKPV VAQGPGPAPG VGSTPPASSS APPATPPTSG APPGSGPGPT
151 PTPPPAVTSA PPGAPPPTPP SSGVPTTPPQ AGGPPPPPAA VPGPGPGPKQ
201 GPGPGGPKGG K**MPGGPKPGG GPGLSTPGGH PKPPR**RGGGE PRGGRQHHPP
251 YHQQHHQGPP PGGPGGRSEE KISDSEGFKA NLSLLRRPGE KTYTQRCR**LF**
301 **VGNLPADITE DEFKR**LFAKY GEPGEVFINK GKGFGFIKLE SRALAEIAKA
351 ELDDTPMRGR QLRVR**FATHA AALSVR**NLSP YVSNELLEEA FSQFGPIERA
401 VVIVDDRGRS TGKGIVEFAS KPAARKAFER CSEGVFLLTT TPRPVIVEPL
451 EQLDDEDGLP EKLAQKNPMY QKERETPTR**F AQHGTFEYEY SQR**WKSLDEM
501 EKQQREQVEK NMKDAKDKLE SEMEDAYHEH QANLLRQDLM RRQEELRRME
551 ELHNQEMQKR KEMQLRQEEE RRRREEEMMI RQREMEDQMR RQREESYSRM
601 GYMDPRERDM RMGGGGAMNM GDPYGSGGQK FPPLGGGGGI GYEANPGVPP
651 ATMSGSMMGS DMRTER**FGQG GAGPVGGQGP R**GMGPGTPAG YGRGREEYEG
701 PNKKPRF

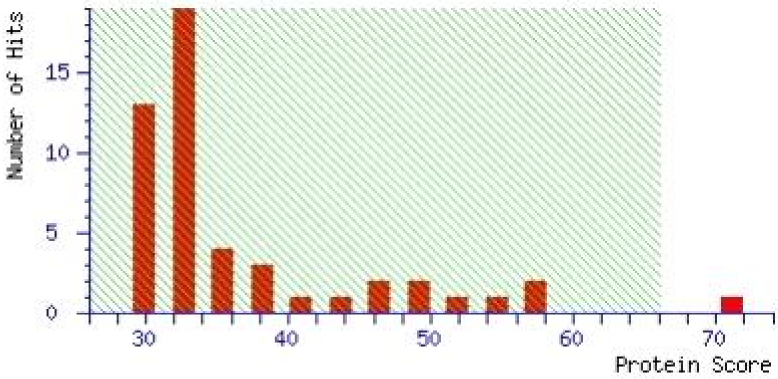

Mascot Score Histogram
Protein score is -10*Log(P), where P is the probability that the observed match is a random event.
Protein scores greater than 66 are significant (p<0.05).
Protein scores are derived from ions scores as a non-probabilistic basis for ranking protein hits.

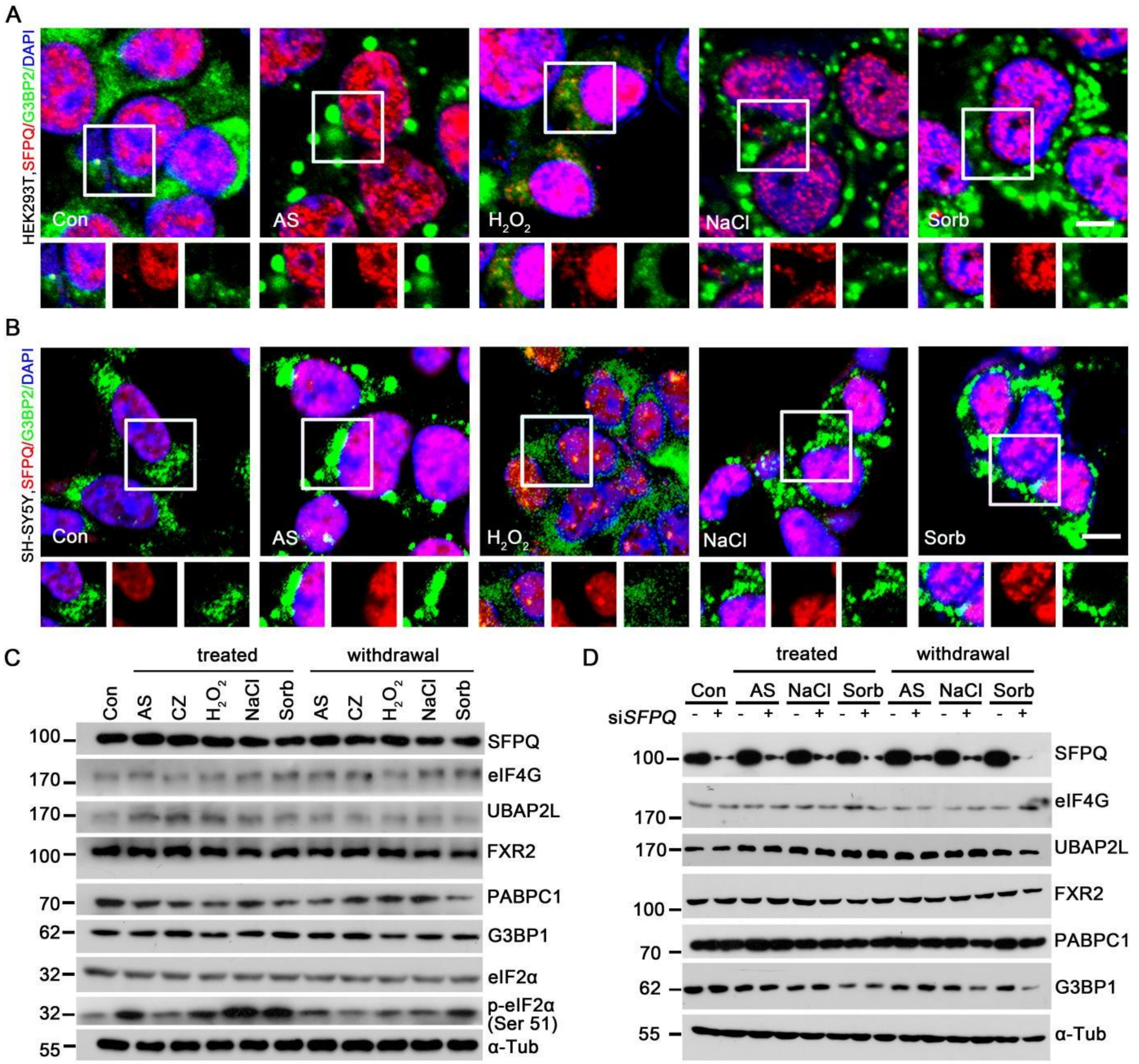

Figure S1. Associations of endogenous cytoplasmic SFPQ with SGs. (A) Co-immunofluorescence of SFPQ (red)/G3BP2 (green) under physiological or stress conditions. HEK293T cells were treated with or without 500 μM sodium arsenite (AS) or 1 mM H_2O_2 for 1 h, or 400 mM sorbitol (sorb) or 200 mM NaCl for 30 min, and then stained for SFPQ (red)/UBAP2L (green). Insets are shown with separated colors. Blue indicates the nuclei counterstained by DAPI. **(B)** Co-immunofluorescence of SFPQ (red)/G3BP2 (green) in SH-SY5Y cells. The treatments were the same as in **(A)**. **(C)** WB analysis of indicated protein levels under stress or recovery conditions. HEK293T cells were treated with the indicated stresses, then were allowed to recover for 1 h before undergoing WB analyses. **(D)** WB analysis of SG protein levels under stress or recovery conditions after SFPQ knockdown. HEK293T cells were transfected with NC- or *SFPQ*-siRNA, followed by treatments and analyses as in **(C).** All scale bars: 5 μm.

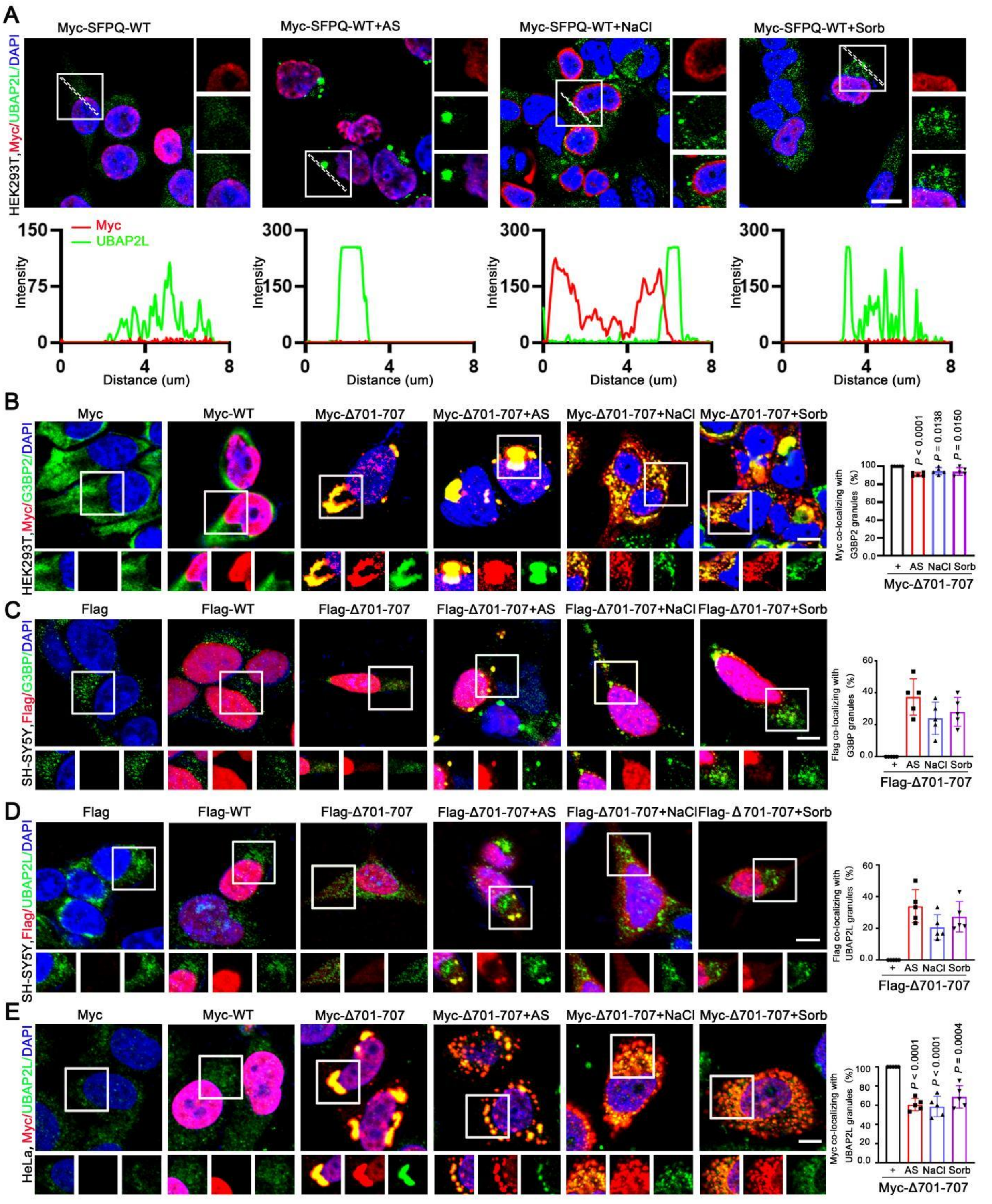

Figure S2. Associations of ectopic cytoplasmic SFPQ with SGs. (A) Co-localization of Myc-SFPQ-WT with UBAP2L in HEK293T cells expressing Myc-SFPQ-WT with or without 500 μM AS for 1 h, or 400 mM sorbitol or 200 mM NaCl for 30 min, followed by co-fluorescence of Myc (red)/UBAP2L (green). Nuclei were counterstained with DAPI (blue), line scans show the related intensity profiles of Myc with UBAP2L. **(B)** Co-localization of SFPQ deletants with G3BP2 in HEK293T cells expressing

Myc empty, Myc-WT or Myc- Δ701-707 with or without 500 μM AS for 1 h, or 400 mM sorbitol or 200 mM NaCl for 30 min, followed by co-fluorescence of Myc (red)/G3BP2 (green). Nuclei were counterstained with DAPI (blue). Percentages of cytoplasmic Myc foci co-localized with G3BP2 granules in Myc^{+} cells . Images are representative of 5 independent frames for each condition, and 20-35 granules were analysed per condition. **(C)** Co-localization of SFPQ deletants with G3BP in SH-SY5Y cells expressing adenoviral Flag, Flag-WT or Flag-Δ701-707, treated as in **(B)**, followed by co-fluorescence of Flag (red)/G3BP (green). Percentages of cytoplasmic Flag foci co-localized with G3BP granules in $Flag^{+}$ cells . Images are representative of 5 independent frames for each condition, and 20-35 granules were analyzed per condition. **(D)** Representative images of UPAB2L in SH-SY5Y cells expressing adenoviral Flag, Flag-WT or Flag-Δ701-707, treated as in **(B)**, followed by co-fluorescence of Flag (red)/UBAP2L (green). Percentages of cytoplasmic Flag foci co-localized with UBAP2L granules in $Flag^{+}$ cells . Images are representative of 5 independent frames for each condition, and 20-35 granules were analyzed per condition. **(E)** Co-localization of SFPQ deletants with G3BP2 in Hela cells expressing Myc empty, Myc-WT, or Myc- Δ 701-707 , treated as in **(B)**, followed by co-fluorescence of Myc (red)/G3BP2 (green). Percentages of cytoplasmic Myc foci co-localized with UBAP2L granules in Myc^{+} cells . Images are representative of 5 independent frames for each condition, and 20-35 granules were analysed per condition. Data are the mean ± standard deviation (SD) and represent three biologically independent experiments. *P* values were determined using one-way ANOVA with Tukey' s multiple comparisons test, comparing Myc-△701-707 to others treatments. All scale bar: 5 μm.

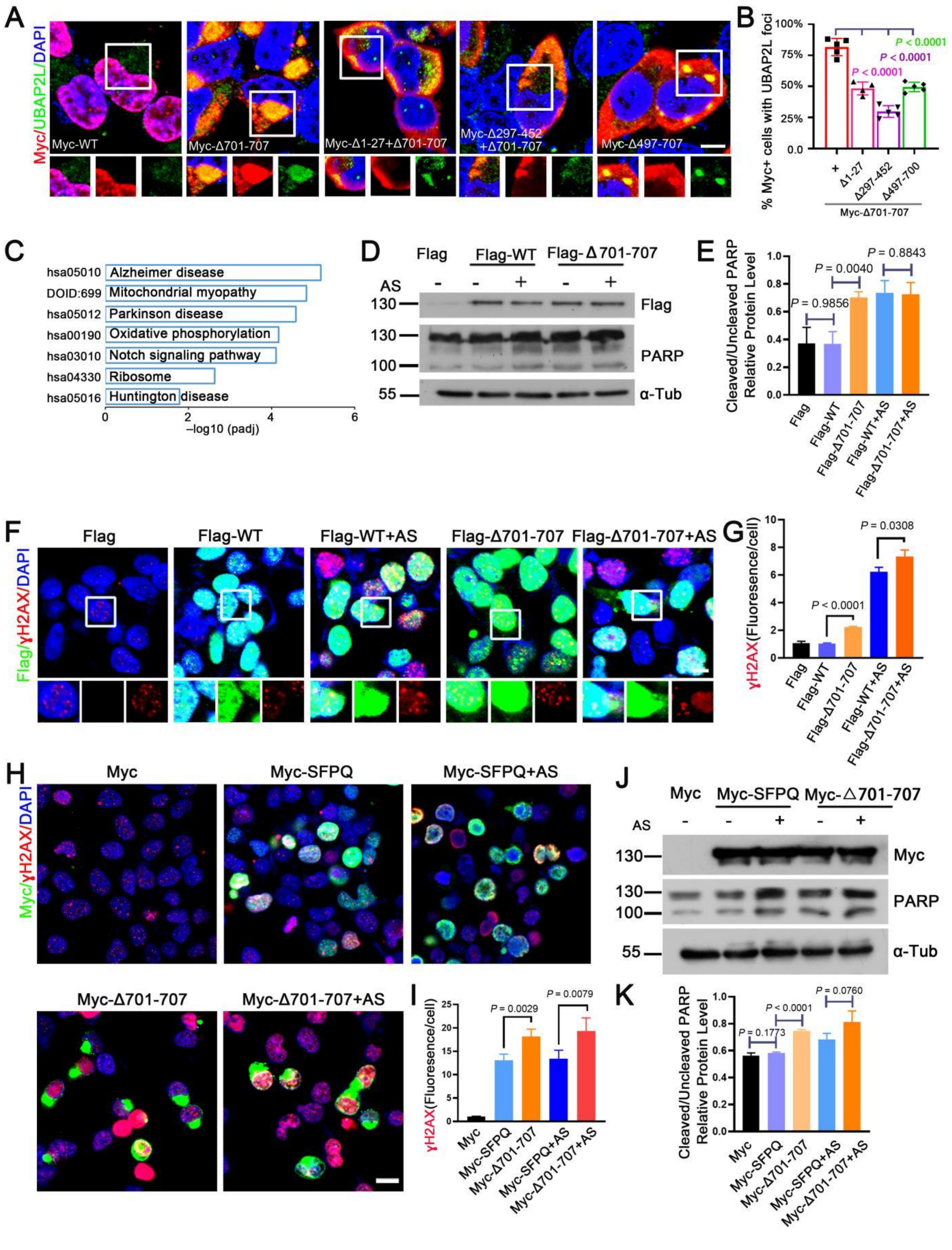

Figure S3. Aberrant cytoplasmic SFPQ induces cell injury. (A, B) Co-localization of SFPQ deletants with UBAP2L in HEK293T cells expressing Myc-WT or indicated deletants double-stained with Myc (red)/ UBAP2L (green). Quantification in **(B)**. **(C)** Bar graphs illustrating top KEGG pathways associated with changed transcripts in SFPQ Δ 701-707 compared with controls. One

significant Disease Ontology (DO) pathway is also showed. **(D, E)** WB analysis of PARP level in SH-SY5Y cells with adenoviral Flag, Flag-SFPQ-WT, or Flag-Δ701-707 with or without 500 μM AS for 1 h. Bars indicate the average level of cleaved-PARP normalized to uncleaved-PARP. Data are the mean±standard deviation (SD) of n = 4 independent repeats. One-way analysis of variance (ANOVA), comparing Flag-WT to Flag-Δ701-707, or Flag-WT+AS to Flag-Δ701-707+AS with Dunnett correction for multiple testing. **(F, G)** γH2AX fluorescence in SH-SY5Y cells with adenoviral Flag, Flag-SFPQ-WT, or Flag-Δ701-707 with or without AS stress, followed by co-fluorescence of Flag (green)/ without AS stress, followed by co-fluorescence of Flag (green)/γH2AX (red). Bars indicate γH2AX intensity in Flag-positive cells in **(G)**, data are the mean ± standard deviation (SD) of n = 3 independent repeats. One-way analysis of variance (ANOVA), comparing Flag-WT to Flag-Δ701-707, or Flag-WT+AS to Flag- Δ 701-707+AS with Dunnett correction for multiple testing. **(H, I)** γH2AX fluorescence in HEK293T cells with Myc-WT or Myc- Δ 701-707 with or without AS stress, followed by co-fluorescence of Myc(green)/γH2AX (red). Bars indicate γH2AX intensity in Myc-positive cells in **(I)**. **(J, K)** WB analysis of PARP level in HEK293T cells with Myc, Myc-SFPQ-WT, or Myc-Δ701-707 with or without 500 μM AS for 1 h. Quantification is showed in **(K)**. Bars indicate the average level of cleaved-PARP normalized to uncleaved-PARP. Data are the mean ± standard deviation (SD) of n = 3 independent repeats. One-way analysis of variance (ANOVA), comparing Myc-WT to Myc-Δ701-707, or Myc-WT+AS to Myc-Δ701-707+AS with Dunnett correction for multiple testing. Scale bar: 5 μm.

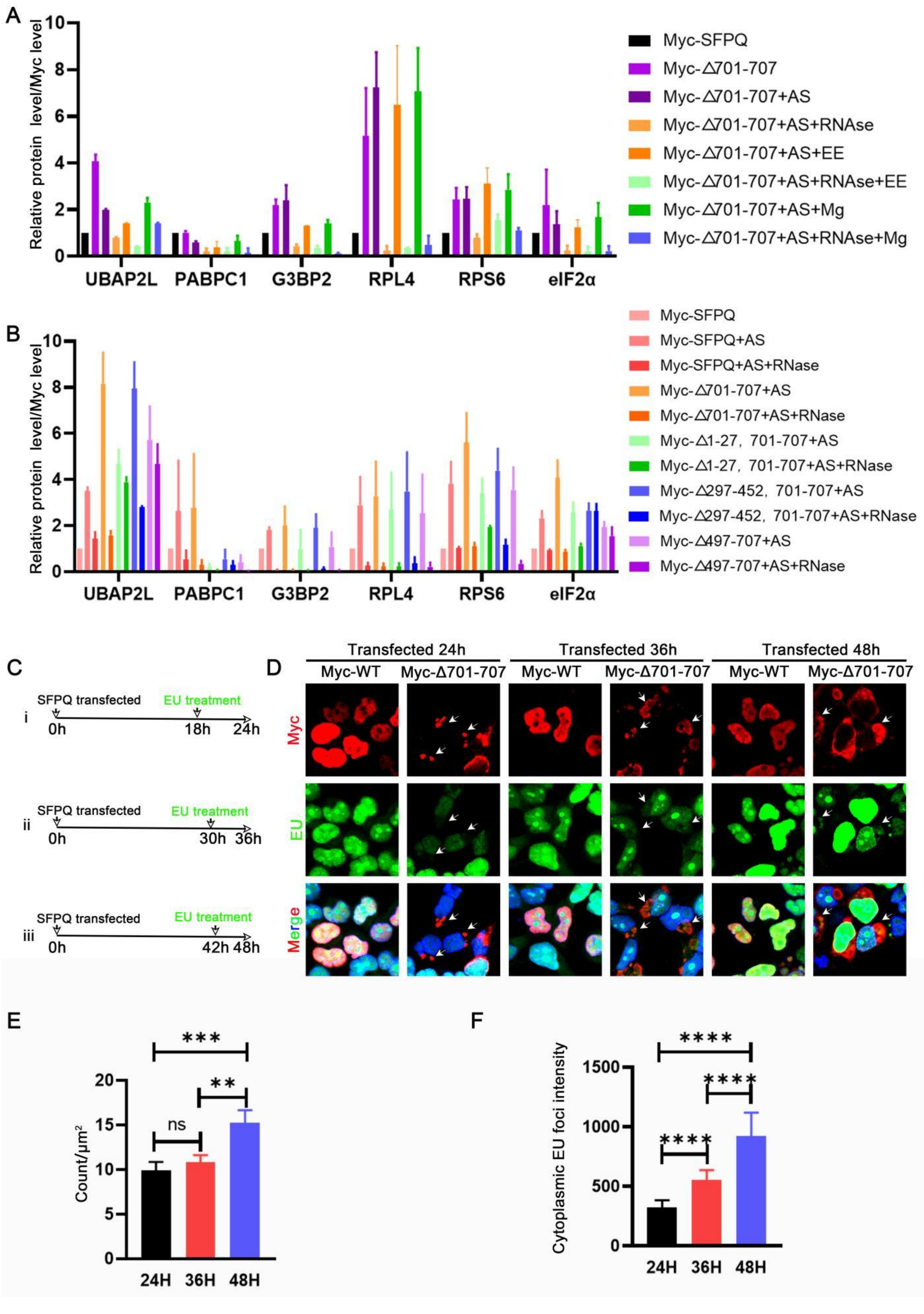

Figure S4. The aberrant global mRNA export depends on the spatiotemporal expression of cytoplasmic SFPQ. (A). Bars indicate the average level of relative proteins to Myc in Figure 3A. Data

are the mean ± standard deviation (SD) of n = 3 independent repeats. **(B)**. Bars indicate the average level of relative proteins to Myc in Figure 3B. Data are the mean ± standard deviation (SD) of n = 3 independent repeats. **(C)** Schematic depicting the experimental design. i) Cells were fixed 6 hours after co-treatment with EU (500 μM, 6 h), following 18 hours of plasmid transfection; ii) Cells were fixed 6 hours after co-treatment with EU (500 μM, 6 h), following 30 hours of plasmid transfection.; iii) Cells were fixed 6 hours after co-treatment with EU (500 μM, 6 h), following 42 hours of plasmid transfection. **(D-F)** Distribution of newborn RNA (EU, green) and its association with Myc (red) in HEK293T cells expressing Myc-WT or indicated Myc-tagged SFPQΔ701-707. Cells were incubated with EU for 24 h,36 h, and 48h, arrows indicate co-localization. Focus size is expressed as fluorescence intensity in μm^2 per focus and is shown in **(E)**, quantification of cytoplasmic EU intensity is shown in **(F)**. Data are the mean ± standard deviation (SD) of n = 4 independent repeats. P values were determined using one-way ANOVA with Tukey's multiple comparisons test, ****P < 0.0001, ***P = 0.0009, **P = 0.0018, ns = 0.1964. All scale bar: 5 μm.

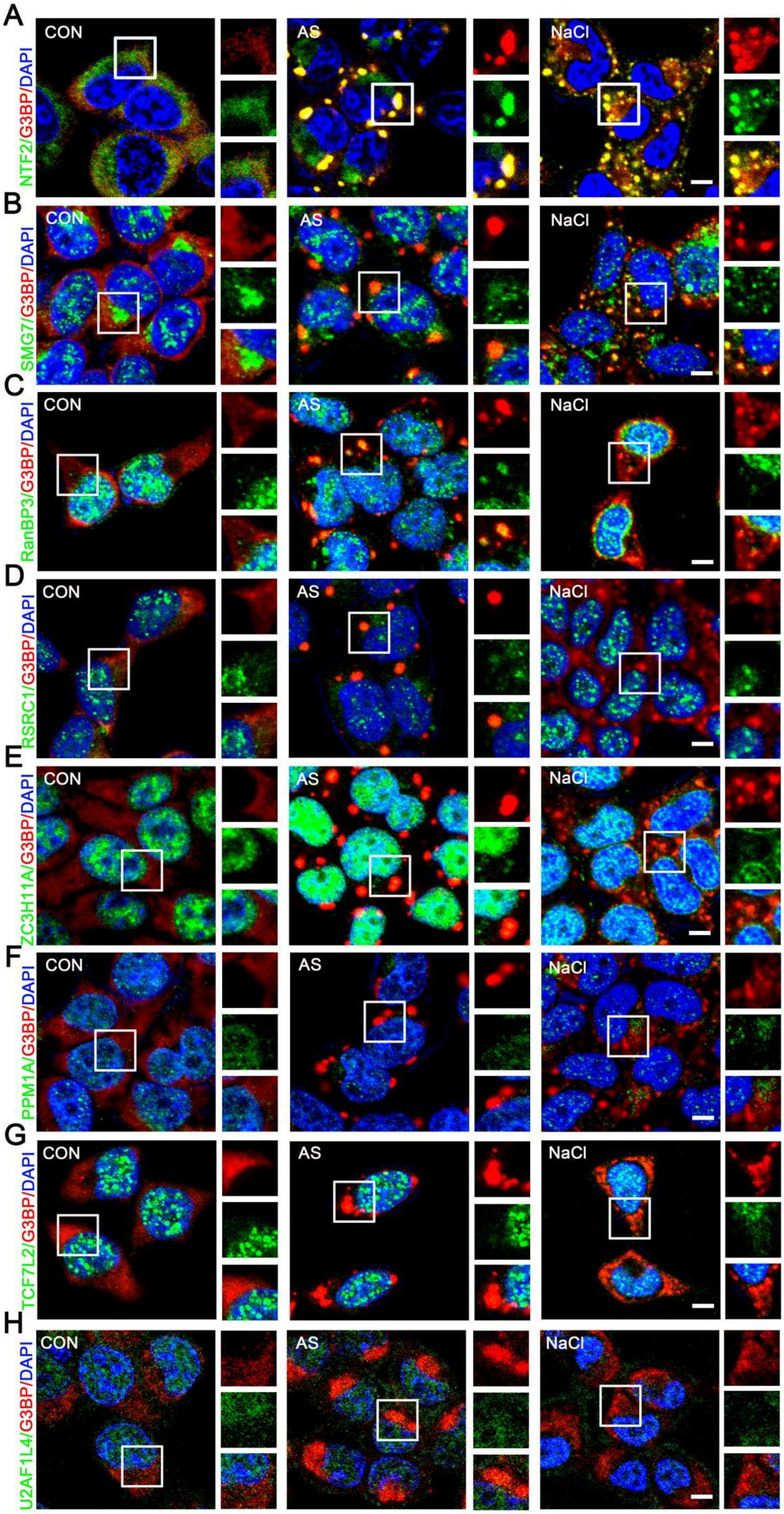

A
NTF2/G3BP/DAPI
CON
AS
NaCl
B
SMG7/G3BP/DAPI
CON
AS
NaCl
C
RanBP3/G3BP/DAPI
CON
AS
NaCl
D
RSRC1/G3BP/DAPI
CON
AS
NaCl
E
ZC3H11A/G3BP/DAPI
CON
AS
NaCl
F
PPM1A/G3BP/DAPI
CON
AS
NaCl
G
TCF7L2/G3BP/DAPI
CON
AS
NaCl
H
U2AF1L4/G3BP/DAPI
CON
AS
NaCl

Figure S5. A portion of nucleocytoplasmic transport proteins translocate into stress granules upon stress. (A-H) Co-IF of nucleocytoplasmic transport proteins with G3BP under physiological or stress conditions. HEK293T cells were treated with or without 500 μM sodium arsenit (AS) or 200 mM NaCl for 30 min, and then stained for G3BP (red) /nucleocytoplasmic transport proteins (green), nuclei were counterstained with DAPI (blue). All scale bar: 5 μm.

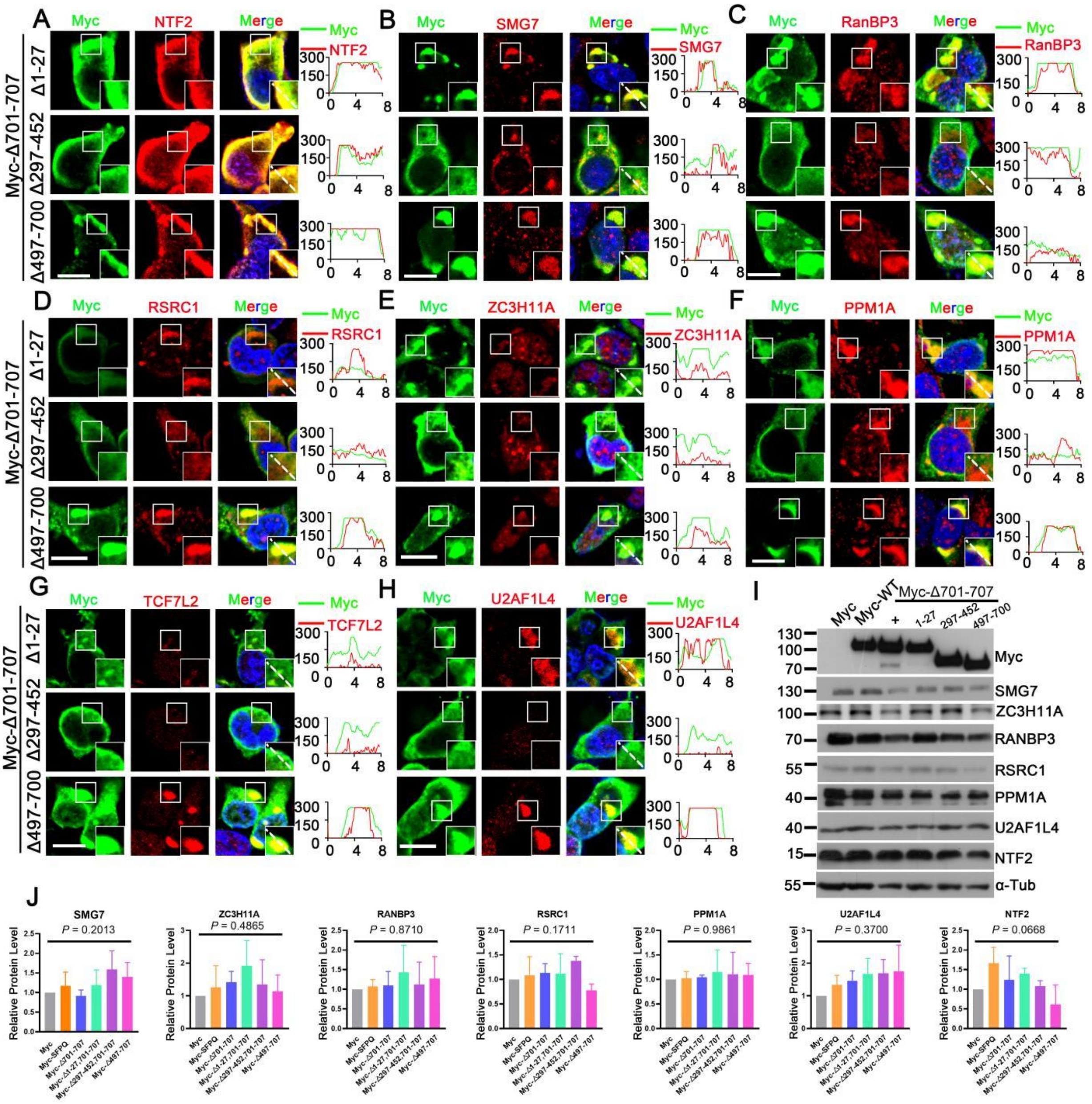

Figure S6. Nucleocytoplasmic transport factors translocate and aggregate into cytoplasm due to mislocation of SFPQ. (A-H) Representative images of nucleocytoplasmic transport factors in HEK293T expressing SFPQ deletants. Line scans show the related intensity profiles of SFPQ with nucleocytoplasmic transport proteins. **(I, J)** WB analysis of indicated protein levels in HEK293T cells expressing Myc empty, Myc-WT, or Myc-SFPQ deletants. **(J)** Bars indicate the average level of indicated protein to α-Tub. Data are the mean ± standard deviation (SD) of n = 3 independent repeats. One-way analysis of variance (ANOVA), not significant ($P > 0.05$). All scale bar: 10 μm.

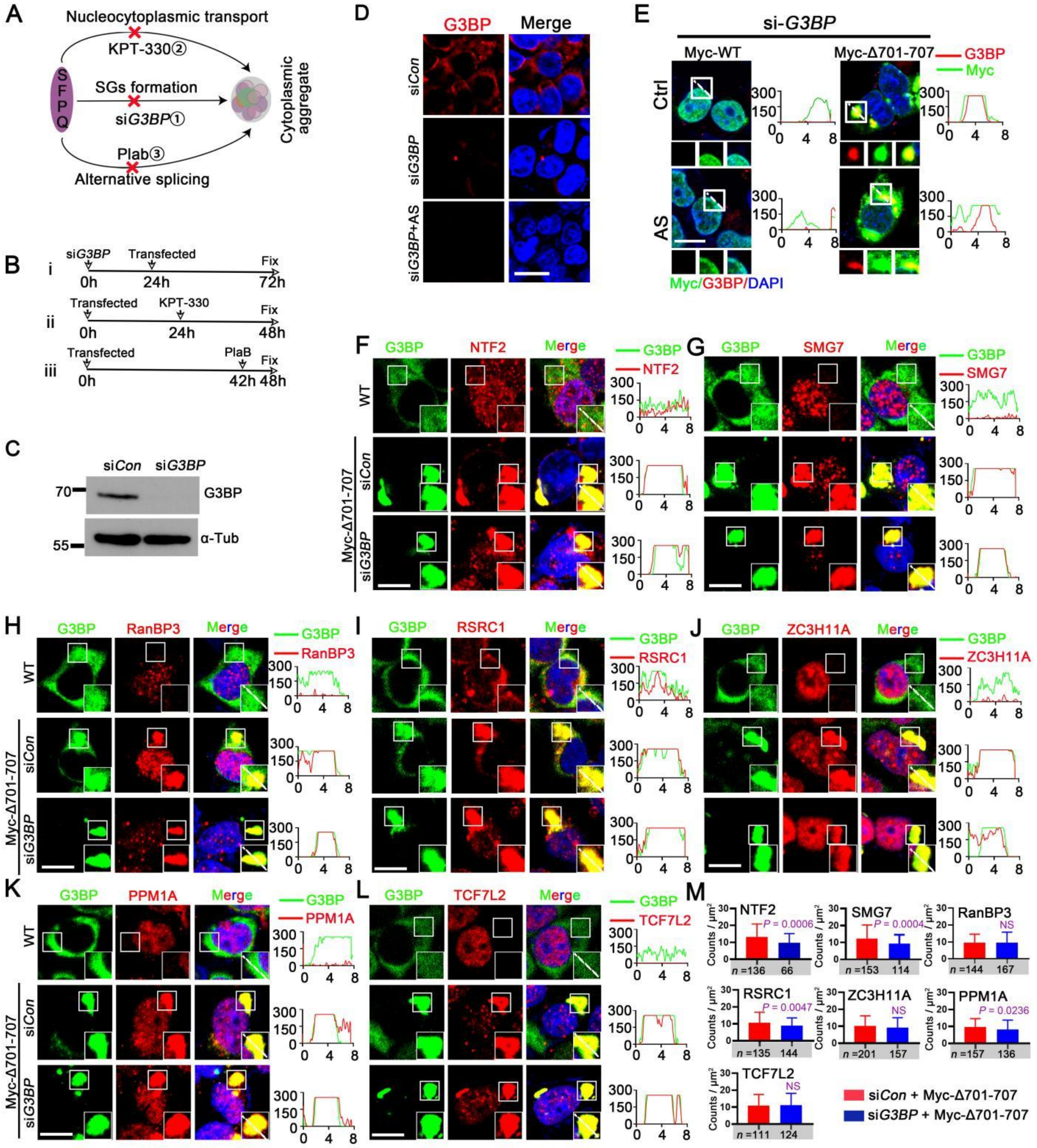

Figure S7. Inhibition of SG nucleation does not alleviate aberrant cytoplasmic SFPQ aggregates. **(A)** Schematic depicting the experimental design: 1) inhibition of SG formation by knocking down G3BP1/2, 2) selective inhibition of nuclear export by KPT-330, and 3) inhibition of alternative splicing by Pladienolide B (PlaB). **(B)** Schematic depicting the experimental design. **(C)** WB analysis of G3BP protein levels in HEK293T cells expressing si*Con* or si*G3BP*. **(D)** Representative images of G3BP in HEK293T cells expressing si*Con* or si*G3BP*, treating with or without 500 μM AS 1h. **(E)** Representative images in HEK293T co-expressing siG3BP (red) and Myc-WT, or Myc-Δ701-707

plasmids (green) treated with or without AS (500 μM). Line scans show the related intensity profiles of SFPQ with G3BP. **(F-M)** Representative images of the association of nucleocytoplasmic transporters with G3BP in HEK293T cells transfected with Myc-WT or Myc-△701-707, with or without si*G3BP*. Line scans show the intensity profiles of G3BP along with nucleocytoplasmic transporters. Focus size is expressed as the fluorescence intensity in μm^2 per focus and is shown in **(M)**. All scale bars: 10 μm. Data are the mean ± standard deviation (SD) of $n = 3$ independent repeats, unpaired Student's t-test, NS, not significant ($P > 0.05$).

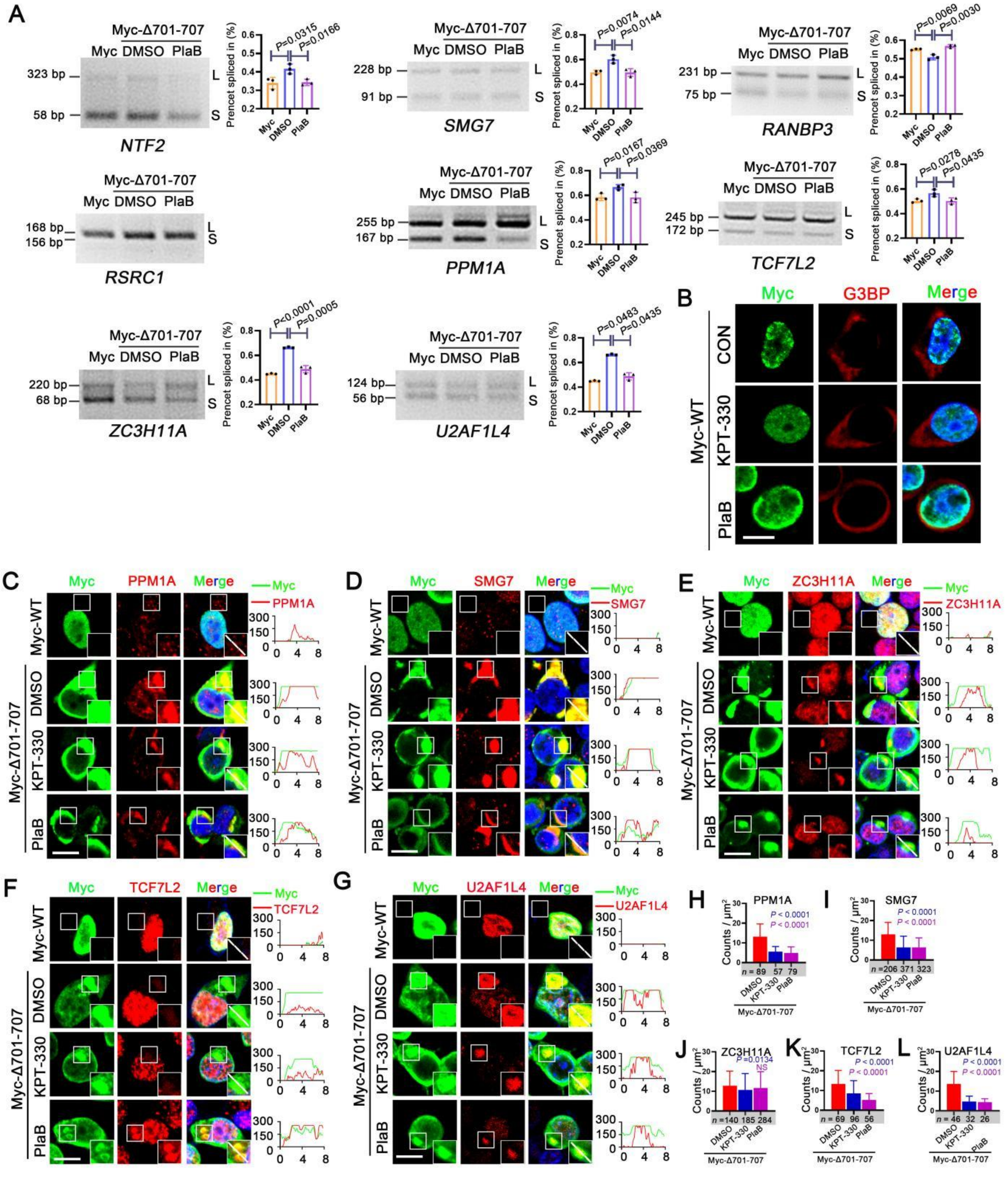

Figure S8. KPT-330 and Plab moderately alleviate the sequestration of nucleocytoplasmic transporters representing Skipped Exon into cytoplasmic SFPQ aggregates. (A) Electrophoresis validating alternative splicing in indicated genes. l, long; s, short. Data were presented as mean ± SD, *n* = 3. One-way analysis of variance (ANOVA), comparing Myc to Myc-Δ701-707+DMSO, or Myc-Δ701-707+DMSO to Myc-Δ701-707+PlaB with Dunnett correction for multiple testing. **(B)** HEK293T cells expressing Myc-WT were treated with DMSO, KPT-330 (3 μM, 24 h), or Plab (10 μM, 6 h), followed by co-IF of Myc and G3BP. **(C-G)** HEK293T cells expressing Myc-WT or Myc-Δ701-707

were treated with DMSO, KPT-330 (3 μM, 24 h), or Plab (10 μM, 6 h), followed by co-IF of Myc and the indicated transporters. **(H-L)** Quantification is expressed as the fluorescence intensity in μm^2 per focus. All scale bars: 10 μm. Data are the mean ± standard deviation (SD) of $n = 3$ independent repeats. Student' s t-test: NS, not significant ($P > 0.05$).

Table S1. Antibodies

Antibody	Vendor	Catalog number	Application	Working Dilution for WB
Mouse anti-Flag tag	Sigma-Aldrich, Shanghai, PRC	F1804	WB, IF, IP	1:5000 (1:200) [1:125]
Rabbit anti-Flag tag	Sigma-Aldrich, Shanghai, PRC	F7425	IF	(1:200)
Mouse anti-Myc Tag	Millipore, Boston, MA, USA	05-724	IF, IP	(1:200)[1:100]
Mouse anti-Myc Tag	Cell Signaling Technology, Shanghai, PRC	9B11	WB,IF	1:2000(1:200)
Rabbit anti-UBAP2L	Bethyl, Montgomery, Alabama, USA	A300-534A	WB, IF	1:2000 (1:100)
Mouse anti-SFPQ	Abcam, Cambridge, MA, USA	ab11825	WB, IF	1:6000(1:200)
Rabbit anti-SFPQ	ABclonal, Shanghai, PRC	A0958	WB, IF	1:6000(1:300)
Rabbit anti-G3BP1	Zen Biosciences, Chengdu, PRC	505342	WB	1:2000
Rabbit anti-G3BP2	Abcam, Cambridge, MA, USA	ab86135	IF	(1:100)
Rabbit anti-G3BP2	ABclonal, Shanghai, PRC	A6026	WB	1:1000
Rabbit anti-PABPC1	ABclonal, Shanghai, PRC	A0516	WB	1:500
Rabbit anti-FXR2	Abcam, Cambridge, MA, USA	ab168852	WB	1:1000
Rabbit anti-RPL4	Proteintech, Wuhan, PRC	11302-1-AP	WB	1:2000
Mouse anti-RPS6	Cell Signaling Technology, Shanghai, PRC	2317	WB	1:4000
Rabbit anti-eIF2α	ABclonal, Shanghai, PRC	A9905	WB	1:1000
Goat anti-eIF4G	Novus Biologicals, Minneapolis, MN, USA	AF4018	WB	1:4000

Rabbit anti-PARP	Cell Signaling Technology, Shanghai, PRC	46D11	WB	1:2000
Rabbit anti-Phospho-H2AX-S139	ABclonal, Shanghai, PRC	AP0687	IF	(1:300)
Mouse anti-α-tubulin	Ray Antibody , Beijing, PRC	RM2007V	WB	1:6000
Mouse anti-U2AF1L3/35(D-4)	SANTA CRUZ BIOTECHNOLOGY, USA	sc-514459	WB,IF	1:2000 (1:50)
Rabbit anti-SMG7	Bioworld Technology, Nanjing, PRC	BS-2112	WB,IF	1:2000 (1:100)
Rabbit anti-G3BP	Zen Biosciences, Chengdu, PRC	R24375	IF	(1:100)
Mouse anti-NTF2	Proteintech, Wuhan, PRC	66063-1-Ig	WB,IF	1:1000 (1:75)
Rabbit anti-RSRC1	Proteintech, Wuhan, PRC	23826-1-AP	WB,IF	1:1000 (1:100)
Rabbit anti-ZC3H11A	Proteintech, Wuhan, PRC	26081-1-AP	WB,IF	1:2000 (1:200)
Rabbit anti-Ranbp3	ABclonal, Shanghai, PRC	A9055	WB	1:2000
Rabbit anti-PPM1A	ABclonal, Shanghai, PRC	A6699	WB,IF	1:2000(1:100)
Rabbit anti-APP	ABclonal, Shanghai, PRC	A17911	IF	(1:200)
Mouse anti-MAP2	Proteintech, Wuhan, PRC	67015-1-Ig	IF	(1:300)
Mouse anti- GFAP	Proteintech, Wuhan, PRC	67015-1-Ig	IF	(1:300)
Rabbit anti-p-eIF2α(S51)	Shanghai, PRC	AP0745	WB	1:1000

Table S2. Primer sequences

Gene	Sense (5'→ 3')	Antisense (5'→ 3')
SFPQ	GCCGAATGGGCTACATGGAT	TCAGTACGCATGTCACTTCCC
PPM1A	CCGAGAACTGTTACTTTCCTCCA	GCCCCTGGGCATTATGCTTT
U2AF1L4	TGCAGGAGAAGTATGGGGAGAT	GGCTGAATGTCGGCTTGTTG
RSRC1	CGGCGCCCTGATCTAAAGAA	GCTCGAGGAGGACCGTCTA
TCF7L2	TCCTTGCCTTTCACTTCCTCC	CAGTCTGTGACTTGGCGTCT
NUTF2	CCATCGTGCCAGCCCC	ATTGCGCCTAGTTGGGTTCT
ZC3H11A	TGGTTTTCTGCTAGTGCTGCT	GCAGGGAATATCCCCAAATCCCTA
RSRC1	CGGCGCCCTGATCTAAAGAA	GCTCGAGGAGGACCGTCTA
SMG7	GGCGGAGGATGAGCCTG	TGACTATCTGCAAGAACATCCAA
GADPH	AACGTGTCAGTGGTGGACCTG	AGTGGGTGTCGCTGTTGAAGT
